# Contrasting Resilience Diagnostics in Route-Preserving Multimodal Transit Hypergraphs

**DOI:** 10.3390/e28090976

**Published:** 2026-09-02

**Authors:** Tian Gao, Ivan Blekanov

**Affiliations:** Faculty of Applied Mathematics and Control Processes, St. Petersburg State University, Saint Petersburg 199034, Russia; st082013@student.spbu.ru

**Keywords:** multimodal transit networks, hypergraph, higher-order networks, potential proximity coupling, route-dependent failure, network resilience

## Abstract

Most multimodal transit studies use pairwise or multilayer graphs that discard route membership. We construct route-preserving bus–metro hypergraphs for 45 Chinese cities. A route-support rule makes node viability depend on the fraction of incident routes that remain viable. Transfer-first attack is the most damaging static strategy in 33 cities. PPCR is associated with greater connectivity retention under random disruption (r=0.807), but with deeper route-support pruning under targeted triggering. Connectivity retention, route-support outcomes, and recovery are only moderately aligned (mean |ρ|=0.52). Three principal components are needed to explain 90% of their variance. Multimodal resilience, therefore, cannot be summarized by one ranking. The hypergraph preserves route-dependent failure as a native model property.

## 1. Introduction

Most resilience studies represent multimodal transit systems as pairwise or multilayer graphs. Stops are linked by adjacency, and transfer stations connect the modes. These representations capture connectivity and intermodal coupling, but they discard route membership. They, therefore, cannot directly express failures that depend on whether the routes serving a stop remain viable. We instead retain routes explicitly and ask whether the same inferred proximity coupling is associated with different resilience outcomes. Transfer nodes may preserve connectivity under diffuse random damage, yet expose shared route dependencies under targeted disruption. Our main question is which structural features matter under each failure regime, and which mechanisms become visible only when route membership is retained.

Previous studies have focused mainly on single-layer rail or metro networks. In these models, stops are nodes and pairwise links connect adjacent stops. Studies across Chinese cities show that attacks on structurally important stations are more damaging than random failures [1,2]. Single-city analyses also find that hubs and transfer stations are especially exposed [3,4,5]. Other work uses structural indices to forecast robustness [6], distinguishes deliberate from random disruption [7], and incorporates passenger weighting, dynamic station importance, or multiple resilience metrics [8,9]. However, a route disappears as an analytical object when it is decomposed into pairwise edges. Route viability can then be evaluated only by reconstructing route membership outside the graph.

Related studies examine cascades in multimodal or interdependent transit systems. They model station- or layer-level dependencies [10,11,12], test alternative representations of multimodal connectivity [13,14], or incorporate redistribution, overload, and delay [15,16,17,18,19,20,21]. This work shows that multimodal vulnerability depends on more than local centrality. Most models nevertheless define coupling between stations, edges, or layers. They can represent node, load, and layer interactions more easily than route-dependent failure. The latter asks whether enough of a node’s routes remain viable after damage.

Higher-order network science provides a direct way to retain routes as objects. In a hypergraph, route membership belongs to the representation itself. Group-dependent processes can, therefore, be defined without reconstructing routes after projection. This distinction matters when a mechanism depends on shared membership rather than pairwise contact [22,23,24]. Methods for reconstructing hypergraphs and detecting higher-order interactions have made these representations more practical [25,26,27]. Recent studies also show that representation choice can change collective dynamics and reveal propagation patterns that pairwise centrality misses [28,29]. In transport, hypergraphs have supported commute estimation and traffic forecasting [30,31,32]. Their use in cross-city resilience analysis remains limited.

Route-level comparative data now make this question tractable. CPTOND-2025 provides standardized bus–metro records at the route and stop levels for many Chinese cities [33]. Large-sample resilience studies nevertheless remain rare. We ask whether potential proximity coupling has the same structural meaning across resilience diagnostics. In particular, can the same inferred coupling align with stronger connectivity retention in one regime and greater dependence in another?

To address these questions, we formalize a route-support mechanism in which node survival depends on the fraction of incident routes that remain viable, placing the model in the family of heterogeneous *k*-core pruning processes [34]. Route-support pruning remains limited under diffuse random damage but becomes more consequential when damage concentrates on shared route memberships.

Our study makes three contributions. First, it introduces a route-preserving multimodal hypergraph and a route-support rule based on surviving route membership. Second, across 45 Chinese cities, potential proximity coupling is associated with different outcomes under different diagnostics. It aligns with greater connectivity retention under random damage, but with deeper route-support pruning under targeted triggering. Third, connectivity retention, route-support outcomes, and recovery are only moderately aligned. Multimodal resilience, therefore, cannot be reduced to one ranking. We use six structurally diverse cities for computationally intensive checks, while estimating the main cross-city associations on the full sample.

## 2. Materials and Methods

### 2.1. Study Scope and Data

The study covers 45 Chinese cities in which both bus and metro systems operate. These cities form the full sample for the cross-city analyses reported in the paper. They are the full set of cities in CPTOND-2025 for which route-level shapefiles were available for both modes at the time of extraction. The sample spans four geographic regions (East, Central, West, and Northeast China) and varies widely in population, network size, and metro maturity. Chuzhou, which has a single metro line, is retained as a structural boundary case in which intermodal coupling is nearly zero.

Transport records come from CPTOND-2025, a standardized national-scale bus–metro vector dataset designed for cross-city comparative analysis with route-level and stop-level operational records. The raw data include stop inventories, route definitions, route–stop incidence tables, and mode labels. From these records we construct city-specific multimodal hypergraph objects. We refer to the resulting measure as the potential proximity coupling ratio (PPCR), rather than an observed PPCR. The main analyses use a 200 m geometric proximity rule; alternative definitions (δ∈{0,100,300} m) are used for robustness checks. This rule identifies structural opportunities for cross-modal proximity, but it does not validate a usable transfer: street barriers, station entrances, grade separation, walking paths, waiting times, fare rules, timetable coordination, and passenger behaviour are not observed. CPTOND-2025 was selected because it provides a common route-level, stop-level, route–stop-incidence and mode-labelled inventory for all 45 cities in the comparison. OpenStreetMap interchange tags and station-level interchange fields are not consistently populated across these cities, whereas CPTOND permits the same construction protocol to be applied cross-sectionally. The dataset does not contain behaviourally validated interchange links, GTFS/NeTEx transfer fields, smart-card transfers, service frequencies, or passenger flows; therefore, the inferred links and all cascade results are structural counterfactuals rather than operationally calibrated observations.

### 2.2. Hypergraph Construction

**Figure 1:**
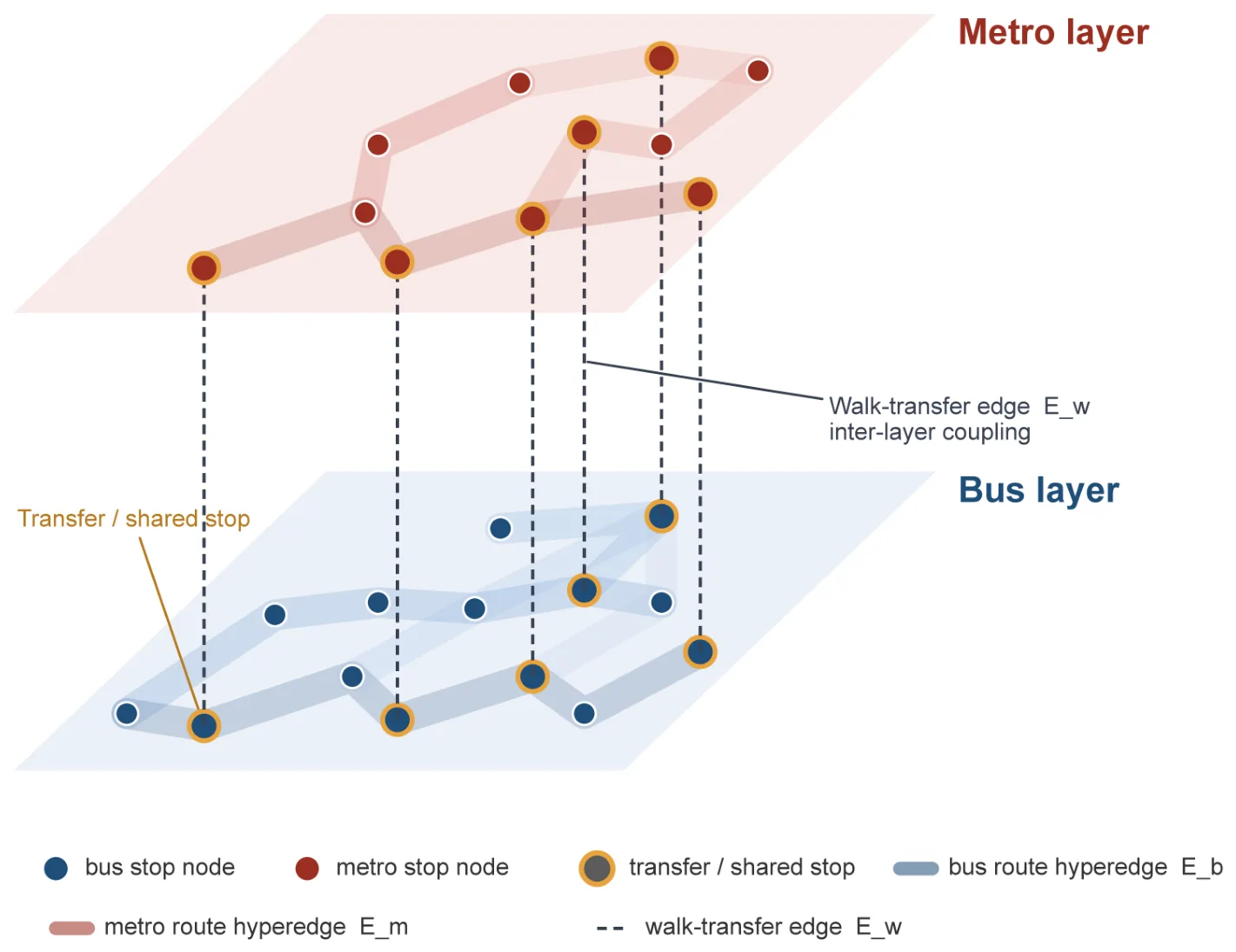
Multimodal transit hypergraph framework. Bus stops ($V_{b}$, blue circles) and metro stations ($V_{m}$, red circles) form the node set *V*; orange rings identify inferred transfer/shared-stop locations. Bus and metro route hyperedges ($E_{b}$, $E_{m}$) retain full route membership; walk-transfer edges ($E_{w}$, dashed lines) connect the two modal subnetworks.

Each city is modeled as a multimodal transit hypergraph (Figure 1) in which routes are retained as explicit group interactions rather than decomposed into dyadic links. Formally,

(1) $$G=(V,E_{b},E_{m},E_{w}),$$

where *V* denotes the set of stop nodes, $E_{b}$ the set of bus route hyperedges, $E_{m}$ the set of metro route hyperedges, and $E_{w}$ the set of walk-transfer edges connecting the two modes. The node set *V* includes both bus stops and metro stations, with shared transfer locations retained as mode-specific nodes linked through $E_{w}$. Route hyperedges in $E_{b}$ and $E_{m}$ are constructed directly from route–stop incidence records. Each route hyperedge is stored as an ordered stop sequence rather than an unordered set. Sequence order is used for directed path projection and consecutive-stop connectivity, whereas route membership is used by the route-support pruning rule. The representation is, therefore, a route-preserving ordered hypergraph with mode-labelled nodes and ordered route hyperedges.

The walk-transfer edge set $E_{w}$ is constructed by a distance-capped union of two matching criteria. Let $V_{b}\subset V$ and $V_{m}\subset V$ denote the bus-stop and metro-station subsets. The name-matched transfer set under threshold δ is

(2) $$E_{w}^{\mathrm{name}}\left(\delta \right)=\left\{\left(v_{b},v_{m}\right)\in V_{b}\times V_{m}\;|\;\mathrm{name}\left(v_{b}\right)=\mathrm{name}\left(v_{m}\right),\;d\left(v_{b},v_{m}\right)\leq \delta \right\},$$

and the distance-based walk-transfer set is

(3) $$E_{w}^{\mathrm{walk}}\left(\delta \right)=\left\{\left(v_{b},v_{m}\right)\in V_{b}\times V_{m}\;|\;d\left(v_{b},v_{m}\right)\leq \delta \right\}\setminus E_{w}^{\mathrm{name}}\left(\delta \right),$$

where d(·,·) is the Haversine great-circle distance. We use the Haversine formula only as a reproducible geographic approximation for stop-to-station distance; at the 100–300 m scale, street-level barriers, entrance locations, grade separation and vertical station structure are much more consequential than Earth curvature. The complete distance-capped transfer edge set is

(4) $$E_{w}\left(\delta \right)=E_{w}^{\mathrm{name}}\left(\delta \right)\cup E_{w}^{\mathrm{walk}}\left(\delta \right).$$

Thus, no same-name pair is retained solely because of its name: every retained candidate must satisfy the same geographic distance cap. Name agreement records the provenance and confidence of the candidate; it does not waive the distance condition. These transfer edges approximate structural walk-transfer opportunities between modes rather than observed transfer volumes or passenger flows. Bus stops and metro stations remain mode-specific nodes even when they share a name or location. Cross-modal transfer edges are, therefore, explicit links between the two node layers. Transfers between routes within the same mode are represented by shared node membership: multiple bus routes or metro lines incident to one node are coupled through that node and do not require an additional transfer edge.

Several derived quantities are defined on this hypergraph. Let $E_{r}=E_{b}\cup E_{m}$ be the set of all route hyperedges. The hyperdegree of node v∈V is the number of routes that include it,

(5) $$d_{H}\left(v\right)=\sum_{e\in E_{r}}1\left(v\in e\right),$$

and the size of route hyperedge $e\in E_{r}$ is s(e)=|e|, i.e. the number of stops on that route. At the city level,

(6) $${\bar{d}}_{H}=\frac{1}{\left|V\right|}\sum_{v\in V}d_{H}\left(v\right),\;\bar{s}=\frac{1}{|E_{r}|}\sum_{e\in E_{r}}s\left(e\right).$$

The potential proximity coupling ratio (PPCR), denoted by $\rho_{\mathrm{ppc}}$, is defined as the fraction of nodes incident to at least one inferred cross-modal proximity edge,

(7) $$\rho_{\mathrm{ppc}}=\frac{\left|\{v\in V:\exists u\in V,\;\left(v,u\right)\in E_{w}\;\mathrm{or}\;\left(u,v\right)\in E_{w}\}\right|}{\left|V\right|},$$

which captures the density of inferred structural proximity coupling independently of system size. It should not be interpreted as a measure of realized passenger transfers or transfer demand.

We calculate connectivity metrics on a directed projection of the hypergraph. These metrics include the largest weakly connected component (LWCC), largest strongly connected component (LSCC), reachability, and efficiency. Each route becomes directed edges between consecutive stops, with both directions retained for bidirectional service. Bus–metro transfer pairs become bidirectional edges. The hypergraph itself is retained for the route-support rule, route-based attack and recovery orderings, and the HER and CLR retention metrics. We also report the cross-mode largest connected component (cross-mode LCC), which measures the largest component containing both bus and metro nodes. Overall LWCC can remain large when one mode stays connected after intermodal links disappear. We, therefore, add two multimodal endpoints. Cross-mode LCC is the fraction of all nodes in the largest weak component containing both modes. Cross-mode reachability is the fraction of sampled ordered bus–metro and metro–bus pairs connected by a directed path. We report both endpoints alongside LWCC in the transfer-edge-removal experiment. A public-transport network need not be strongly connected. Terminals, one-way sequences, city-boundary truncation, and asymmetric service can create separate directed components even when the undirected infrastructure is connected. We, therefore, use LWCC as the primary general-connectivity endpoint. Strong connectivity and directed reachability are complementary diagnostics. In the transfer-edge-removal experiment, however, cross-mode LCC and cross-mode reachability are the co-primary endpoints for multimodal integration because LWCC can remain high after the modes become disconnected.

### 2.3. Resilience Metrics and Disruption Protocols

The two-stop minimal-connectivity condition is a structural convention. It does not claim that a real route can operate with only two stops. Likewise, τ is a support threshold, not a physical station-closure probability. If two of three metro lines are suspended, the model records a loss of route support; it does not assert that the station physically closes. This rule also differs from an epidemic model. Epidemic models usually update nodes through infection probabilities and recovery transitions. Our rule updates nodes deterministically from route viability and each node’s viable-route fraction. It has no epidemiological transmission or recovery state. For static robustness, the main metric is the area under the curve (AUC) of the largest weakly connected component (LWCC) fraction over increasing disruption intensity. We also report the largest strongly connected component (LSCC), the fraction of reachable ordered node pairs, average directed efficiency, and three retention indices: node retention rate (NRR), hyperedge retention rate (HER), and cross-layer retention rate (CLR). This multi-metric view follows a growing transit-resilience literature that evaluates network performance along more than one structural dimension rather than relying on a single scalar robustness index [35,36].

For route-support cascades, we summarize cascade breadth as the fraction of the system lost by the time propagation stops and cascade depth as the number of rounds before convergence. Recovery performance is summarized by the recovery AUC of the LWCC fraction and by

(8) $$t_{90}=\min\left\{q\in \left[0,1\right]:R_{\mathrm{rec}}\left(q\right)\geq 0.9\;R_{0}\right\},$$

the fraction of nodes that must be restored to reach 90% of baseline performance.

We evaluate four families of disruption. The random disruption family serves as the baseline. Seven settings are run (all-mode, bus-only, and metro-only node removal; all-mode, bus-only, and metro-only route removal; and transfer-edge removal), each with 100 Monte Carlo repetitions per attack-fraction level.

The targeted disruption family uses deterministic attack orders: T1 (by hyperdegree), T2 (by betweenness), T3 (transfer nodes first), T4 (by route size), T5 (by route betweenness), and T6 (by sum of hyperdegrees at transfer endpoints).

The disruption experiments are structural counterfactuals, not reconstructions of a specific incident. Node removal deletes the node from every incident route sequence. The route is neither split nor allowed to skip the failed stop. Route removal marks only the selected route as inactive; its nodes and other routes remain. Transfer-edge removal deletes only the cross-modal connection and retains both endpoints. It represents a controlled loss of interchange connectivity rather than a uniquely identified accident.

The cascade family models secondary structural propagation under the route-support rule. Scenarios C1–C3 start from random node damage at tolerance τ∈{0.2,0.4,0.6}; C4 uses targeted initial triggering; C5 starts from random transfer-edge damage. This family is designed to complement two existing lines of multimodal cascade modelling. One line emphasizes coupled map lattices and load redistribution [15,16,17,20,21]; the other emphasizes interdependent layers, coupling strength, and multimodal propagation structure [10,11,12]. The present framework adds a route-membership-dependent failure mechanism in a route-preserving representation. Let $D_{0}\subset V$ be the initially damaged nodes and τ∈(0,1] the tolerance threshold. The initial survivors are $V_{0}^{\mathrm{alive}}=V\setminus D_{0}$. At each round *t*, the set of routes still serving node *v* is

(9) $$E_{r}\left(v,t\right)=\left\{e\in E_{r}\left(v,0\right)\;|\;\left|e\cap V_{t}^{\mathrm{alive}}\right|\geq 2\right\},$$

and the fraction of *v*’s original routes still intact is

(10) $$\phi_{r}\left(v,t\right)=\frac{|E_{r}\left(v,t\right)|}{|E_{r}\left(v,0\right)|}.$$

Node *v* fails at round t+1 if $\phi_{r}\left(v,t\right)<\tau$. The new failures are

(11) $$F_{t+1}=\left\{v\in V_{t}^{\mathrm{alive}}\;|\;\phi_{r}\left(v,t\right)<\tau \right\},$$

and survivors update synchronously:

(12) $$V_{t+1}^{\mathrm{alive}}=V_{t}^{\mathrm{alive}}\setminus F_{t+1}.$$

For transfer-based cascades (C5), the same logic applies but using the fraction of surviving transfer edges,

$$\phi_{w}\left(v,t\right)=\frac{|E_{w}\left(v,t\right)|}{|E_{w}\left(v,0\right)|}.$$

The cascade ends when no new nodes fail ($F_{t^{*}}=Ø$), giving the collapse fraction

(13) $$\Gamma =1-\frac{|V_{t^{*}}^{\mathrm{alive}}|}{\left|V\right|}.$$

The route-support rule is a stylized pruning rule, not a calibrated operational failure model. It captures route-dependent structural loss but omits observed service failures, passenger behaviour, demand, frequency, capacity, and recovery time. We, therefore, treat τ as a sensitivity parameter. At τ=0.2, a node fails after losing more than 80% of its routes; at τ=0.6, failure occurs after a 40% loss. Section 3.6 and Supplementary Tables S16 and S17 test a finer tolerance grid and alternative route-viability conditions. The outcomes describe relative structural vulnerability, not realized operational disruption. Unlike neighbour-fraction, load-capacity, and station-coupling rules [10,11,15], our rule evaluates survival from incident routes rather than surviving neighbours or transferred load.

The condition $|e\cap V_{t}^{\mathrm{alive}}|\geq 2$ defines the minimal-connectivity condition. It is not an operational definition of route continuity. Real routes differ in length, ridership, frequency, capacity, and redundancy. We, therefore, test proportional alternatives, $|e\cap V_{t}^{\mathrm{alive}}\left|\geq \max(2,\lceil \alpha |e|\rceil \right)$. Stricter values of α increase cascade depth, collapse breadth, and connectivity loss. Across the six outcome-informed exploratory cities, the curve-averaged collapse fraction rises from 0.451 under the two-stop condition to 0.683 at α=0.7. Mean depth rises from 1.68 to 3.63. The direction is consistent, but city rankings are not invariant across all α values. We, therefore, emphasize group-level structural comparisons rather than definitive operational rankings.

The protocol is intentionally topological. A failed node is removed from every route containing it; routes are not split and do not skip the stop. A route becomes non-viable when its surviving-stop count fails the selected minimal-connectivity condition. Its remaining stops are not removed directly. They can fail in a later synchronous round if their viable-route fraction falls below τ. Transfer-edge removal is separate: the transfer pair is deleted while its endpoints remain. These conventions define controlled counterfactual experiments. Accordingly, “node failure” means loss of structural support in the model, not necessarily physical station closure.

The two-stop condition in Equation (9) is the minimal-connectivity convention used in this study. If exactly two ordered stops remain, the route is retained as a minimal surviving segment connecting two distinct stop nodes. If fewer than two stops remain, the route cannot provide a connecting relation in the projected network and is marked non-viable. This convention prevents a single surviving stop from being counted as a functioning route; it is not a claim that a real service can operate with exactly two stops.

Equation (11) applies the route-support mechanism at the node level. A node is marked structurally unsupported when the fraction of its incident routes that remain viable falls below τ, and the removal is applied in the next synchronous round. For example, if three metro routes serve a station and two become non-viable, the viable-route fraction is 1/3. The station is removed under this rule when τ>1/3 and remains supported when τ≤1/3. This represents loss of route-based structural support under a counterfactual pruning rule, not an assertion that a physical station must close whenever two of three lines are suspended.

The cascade is, therefore, not an epidemic model applied verbatim to transportation. Epidemic models generally use transmission probabilities, infection states, and recovery transitions. Here, route viability is binary, node support is a deterministic fraction of viable incident routes, and all newly identified failures are updated synchronously. The state variables, transition mechanism, and interpretation are consequently different.

This rule belongs to the family of *k*-core pruning processes, originally characterized by Dorogovtsev et al. [34]. A node *v* survives round *t* if at least

(14) $$k\left(v\right)=\left\lceil \tau \cdot d_{H}\left(v\right)\right\rceil$$

of its incident hyperedges are still viable. Because k(v) scales with the initial hyperdegree $d_{H}\left(v\right)$, high-degree nodes must retain more routes in absolute terms even though all nodes share the same relative tolerance τ. The rule differs from node-based *k*-core pruning in two ways. First, survival depends on viable hyperedges rather than surviving neighbours. Second, heterogeneity is defined by hyperdegree, the number of routes serving a node, rather than pairwise degree. This makes route overlap explicit and illustrates how representation determines which processes can be defined directly [22,23,28,29].

The recovery family tests restoration order after 50% initial damage. REC1 uses random recovery after random damage. REC2 prioritizes hyperdegree, and REC6 uses a one-step marginal-LWCC heuristic. These three strategies use the same damage realizations. At each step, REC6 restores the failed node that gives the largest immediate LWCC gain, assuming complete knowledge of the damage state. REC3–REC5 combine structure-aware recovery with matched targeted damage. Random experiments use 100 Monte Carlo repetitions with reproducible seeds. More expensive model comparisons use 50 repetitions, and counterfactual checks use 20. Section 3.6 reports convergence diagnostics. Related work covers rail repair sequencing, station restoration, and uncertainty- or risk-based infrastructure recovery [37,38,39,40,41,42,43,44,45,46,47,48].

As a baseline, we also implement a Buldyrev-style interdependent cascade between bus and metro [11]. Nodes are partitioned by mode. In each round, a surviving node is removed if it lies outside its layer’s largest connected component. Failure then propagates through transfer pairs: failure of one endpoint removes its coupled endpoint in the other mode. Updates continue synchronously until no new failures occur. This model has no tolerance threshold. Its dynamics depend on within-layer connectivity and cross-layer dependency links. We use 10% initial random damage and 50 repetitions per city, matching the other model comparisons.

Table 1 summarises all disruption, cascade, and recovery scenarios used in the study.

### 2.4. Clustering and Analysis-Set Selection

For reproducibility, the stop-count descriptor is the total multimodal node count ($n_{\mathrm{nodes},\mathrm{total}}$), and the route-count descriptor is the total route-hyperedge count ($n_{\mathrm{hyperedges},\mathrm{total}}$); both are log-transformed before modelling. For the analyses added during revision, we froze a structure-only replication set selected from six pre-disruption descriptors: system size, route count, PPCR, modal composition, hyperdegree, and route size. It comprises Guangzhou, Shanghai, Tianjin, Xuzhou, Zhengzhou, and Urumqi. The original 13-feature clustering included resilience outcomes; its six cities (Guiyang, Chengdu, Chuzhou, Qingdao, Shijiazhuang, and Fuzhou) are, therefore, retained only as outcome-informed exploratory examples. The primary cross-city analyses use all 45 cities, and the structure-only set provides the confirmatory replication of the computationally intensive factorial comparison. Hierarchical clustering selects cities for computationally intensive analyses; it is not used to estimate the main cross-city associations. Those associations use all 45 cities. The descriptive clustering combines 13 standardized features. Six describe pre-disruption structure: log stop count, log route count, PPCR, metro-node ratio, average hyperdegree, and average route size. Seven summarize resilience: LWCC AUC under two attacks, cascade depth under random and targeted triggering, targeted collapse fraction, and recovery AUC under two strategies. We standardize each feature as $z_{ij}=\left(x_{ij}-\mu_{j}\right)/\sigma_{j}$.

Adding resilience outcomes separates some cities that have similar size and PPCR but different route overlap. Using only the six structural features produces a different partition and representative set, while leaving the main robustness patterns similar. We apply Ward linkage and choose K=6 to obtain broad representative coverage. Silhouette scores remain low, which indicates a continuum rather than sharply separated city types. We, therefore, use the centroid-nearest cities as examples and do not interpret the clusters as natural classes. The Adjusted Rand Index (ARI) compares the two partitions: 1 indicates identical partitions, 0 indicates chance-level agreement, and negative values indicate less agreement than expected by chance [34].

The representative city of each cluster is the member closest to the centroid:

(15) $$i_{c}^{*}=\arg\min_{i\in C_{c}}{∥z_{i}-m_{c}∥}_{2}.$$

### 2.5. Robustness and Statistical Design

**Figure 2:**
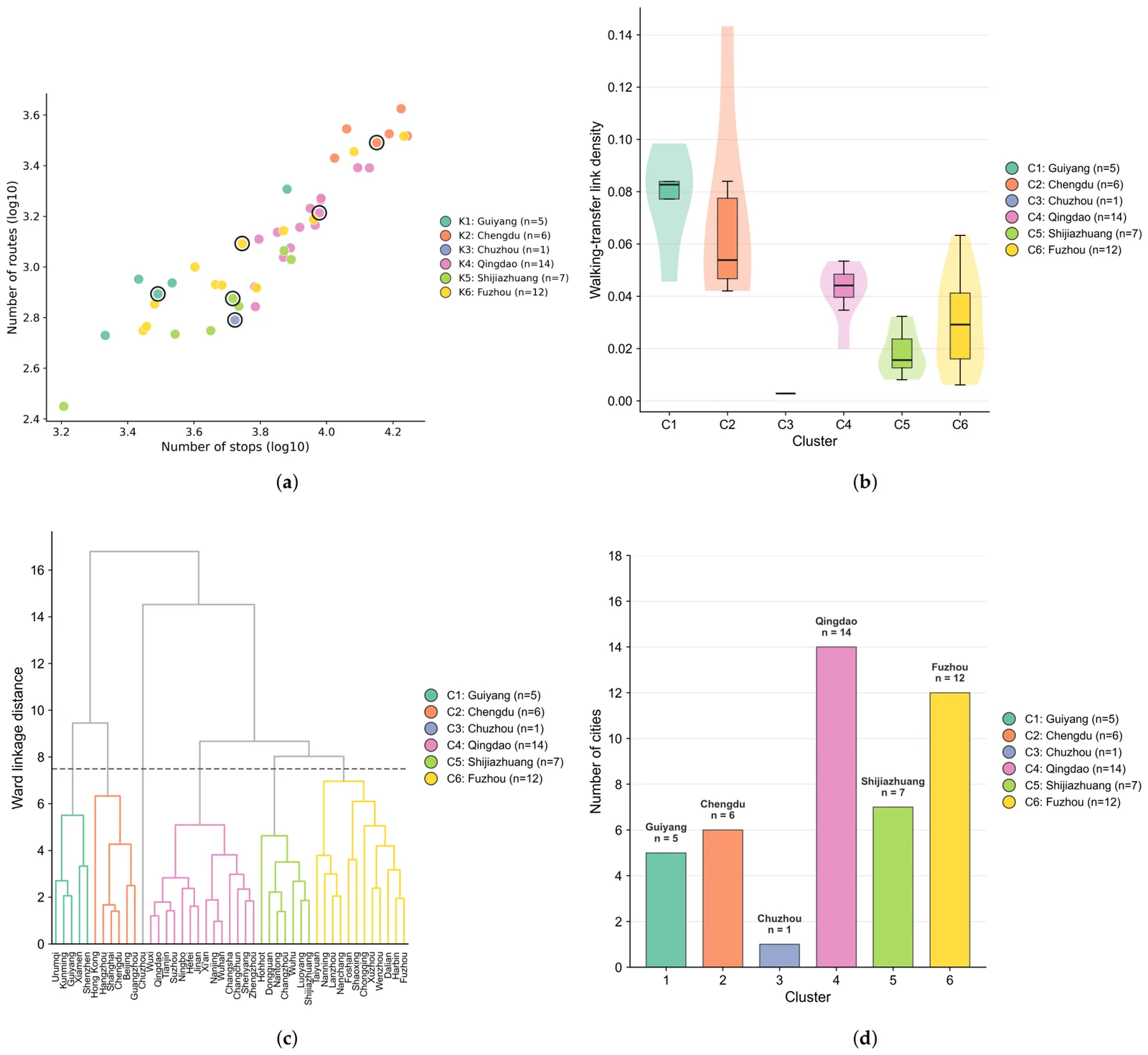
Structural heterogeneity and outcome-informed clustering of 45 cities. (**a**) $\log_{10}$ stops versus $\log_{10}$ routes; colors denote clusters, and black rings mark centroid-nearest representatives. (**b**) Cluster distributions of walking-transfer link density (violin and box plots). (**c**) Ward dendrogram based on 13 standardized structural and resilience features. Branch colors match the legend symbols; gray branches denote mergers above the six-cluster cut (dashed line). C3 is a singleton and has no within-cluster branch. (**d**) Cluster sizes; annotations give the centroid-nearest representative and number of cities.

Figure 2a reports pairwise correlations. By contrast, the VIF values are multivariable diagnostics from the joint six-predictor design matrix with an intercept. They, therefore, need not correspond to any single pairwise correlation.

Robustness across network versions is assessed on the six outcome-informed exploratory cities using relative drift,

(16) $$\mathrm{drift}=\frac{\max_{k}m_{k}-\min_{k}m_{k}}{{\mathrm{median}}_{k}\left(m_{k}\right)},$$

where $m_{k}$ is the metric value under network version k∈{1,2,3,4}. In the outcome-informed exploratory-city threshold analyses, we additionally summarize threshold-like transitions by the fitted midpoint $f_{c}\left(\tau \right)$ and the transition width 1/k of the logistic fit. We measure cross-version ranking stability with Spearman rank correlation. Monte Carlo convergence is checked by varying repetitions over {30,50,100,200} and computing the relative deviation from the 200-repetition reference,

(17) $$\delta_{i}\left(n\right)=\frac{\left|{\hat{A}}_{i}\left(n\right)-{\hat{A}}_{i}\left(200\right)\right|}{{\hat{A}}_{i}\left(200\right)}.$$

We quantify cross-city associations with Pearson and Spearman correlations. Log stop count and log route count are strongly collinear (VIF >400). The main regressions, therefore, use log stop count, PPCR, and metro-node ratio; all three VIFs are below 2.5. Five-fold cross-validated LASSO frequently selects variables in this reduced specification, but does not identify it uniquely. We also estimate a six-predictor model that adds log route count, average hyperdegree, and average route size (Supplementary Tables S2 and S3). The city is the unit of analysis (n=45). For paired Wilcoxon tests, we report rank-biserial correlation, $r_{b}=1-2U/\left(n_{1}n_{2}\right)$. For paired *t*-tests, we report Cohen’s $d=\bar{\Delta }/s_{\Delta }$. Leave-one-out cross-validation evaluates the three-predictor OLS models, with $Q_{\mathrm{LOO}}^{2}=1-\sum_{i}{\left(y_{i}-{\hat{y}}_{-i}\right)}^{2}/\sum_{i}{\left(y_{i}-\bar{y}\right)}^{2}$. We apply principal component analysis (PCA) to five outcomes: LWCC AUC under random and transfer-first attack, cascade depth, collapse breadth, and recovery AUC. These are cross-sectional associations, not causal estimates. We treat cities as independent observations, although residual regional dependence may remain. We, therefore, interpret individual *p*-values cautiously, report false-discovery-rate checks, and repeat the main analyses without Chuzhou.

The choice of $\log_{10}\left(\mathrm{stops}\right)$ over $\log_{10}\left(\mathrm{routes}\right)$ as the composite size proxy is guided by parsimony and is compatible with the LASSO resampling results, rather than reflecting substantive privilege: the two variables are nearly collinear and capture the same underlying dimension, overall system size. The resampling frequencies show that predictor selection is not unique.

During the experimental stage, we selected three primary contrasts for uncertainty analysis. H1 tests whether PPCR is positively associated with LWCC AUC under random node removal. H2 tests whether PPCR is positively associated with route-support cascade depth. H3 tests whether targeted triggering produces greater depth than random triggering at τ=0.2. We evaluated all three with 5000 city-level bootstrap resamples. The intervals measure sample stability; they are not causal or population-level guarantees.

For H1, the bootstrap estimate was r=0.807 with a 95% interval of [0.688,0.895]. For H2, it was r=0.589 with an interval of [0.289,0.778]. For H3, targeted minus random depth was 0.475 units, with an interval of [0.380,0.577]. In 2000 cross-validated LASSO resamples, PPCR selection frequencies ranged from 0.915 to 0.999. Several size and route-overlap variables were also selected often. The six-predictor model increased in-sample $R^{2}$ by 0.085–0.325 (Supplementary Tables S20–S22). Thus, the main associations are stable under resampling, but predictor selection is not unique.

To assess whether residual geographic dependence biases the cross-city associations, we computed Moran’s *I* on the OLS residuals of the main connectivity-retention regression (LWCC AUC under random node removal regressed on PPCR) using an inverse great-circle distance weight matrix row-standardised across all 45 cities. The test statistic was I=0.020 (p=0.673, 999 permutations), providing no evidence of significant spatial autocorrelation at conventional thresholds. Moran’s *I* was similarly non-significant for the cascade-depth, collapse-breadth, and recovery-AUC residuals (all p≥0.15; Supplementary Material, Table S14). These checks indicate that the reported cross-city associations are not driven by geographic clustering of the 45-city sample.

## 3. Results

### 3.1. Structural Heterogeneity and Exploratory City Trajectories

We organize the cross-city results around three resilience dimensions, static connectivity retention, route-support pruning, and recovery. The analyses of associations and attack rankings use all 45 cities. We use the original six outcome-informed cities only to illustrate disruption trajectories; they are exploratory examples rather than a confirmatory sample. The 45 cities span a broad structural range (Figure 2). Log-transformed stop counts range from 3.21 to 4.24, log route counts from 2.45 to 3.63, and PPCRs from 0.003 to 0.143. The sample includes large hub-dominant systems, compact high-PPCR systems, sparse long-route systems, and one clear low-coupling outlier.

The six centroid-nearest cities from the outcome-informed partition are Guiyang, Chengdu, Chuzhou, Qingdao, Shijiazhuang, and Fuzhou. They provide exploratory test cases for intensive analyses. They separate mainly along PC1 (44.1%, dominated by system size) and PC2 (17.7%, dominated by transfer density). Together they occupy all four reduced-space quadrants (Supplementary Figure S1b–d). Table 2 summarizes their structural contexts.

Chuzhou is a boundary case, not a common city type. It forms a singleton cluster for every *K* from 3 to 6. Its nearest neighbour, Xuzhou, lies 8.4 standardized units away, about four times the typical within-cluster distance. Chuzhou has the lowest PPCR and metro-node ratio (both 0.003; the next-lowest value is 0.006 in Shaoxing; Supplementary Table S1). We retain it as an example of a system with almost no intermodal coupling.

The six outcome-informed exploratory cities show how structural differences translate into distinct resilience patterns. Under static attack, hub-dominant systems such as Chengdu tolerate random removal relatively well but deteriorate quickly under targeted attack, while Chuzhou declines steeply even under random disruption because its metro layer provides almost no structural backup. Route-support cascade trajectories reveal a more subtle contrast: cities with similar initial losses can differ sharply in secondary structural propagation depending on route overlap and transfer concentration. Recovery trajectories show a consistent advantage for structure-aware recovery, with the largest gains in hub-dominant and high-PPCR systems. The full exploratory attack, cascade, and recovery trajectories for these six cities are provided in the Supplementary Material (Figures S5–S7).

### 3.2. Transfer-First Attack Is Associated with the Lowest Static Retention in Most Cities

**Figure 3:**
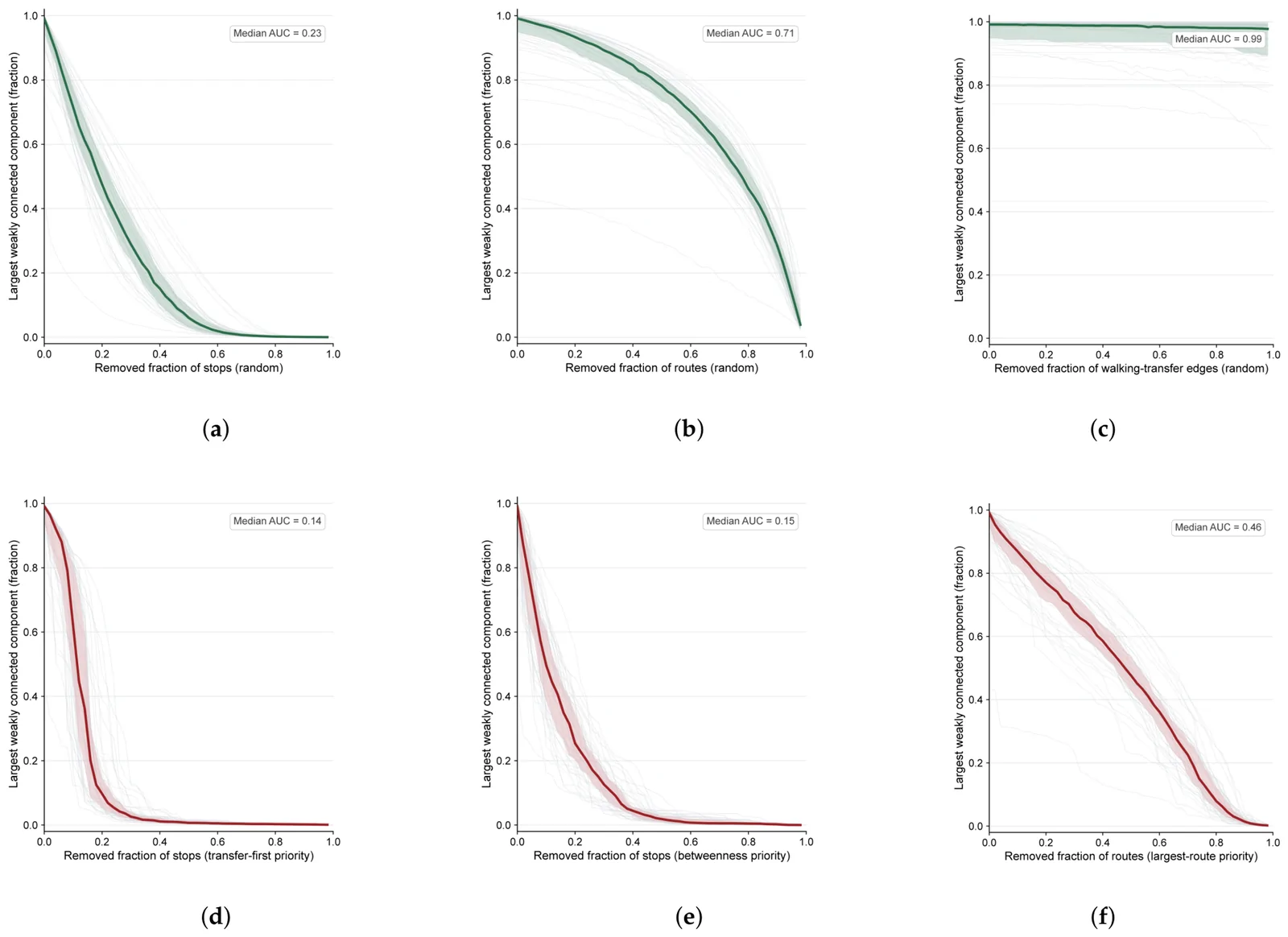
Cross-city connectivity retention under attack. (**a**–**c**) LWCC AUC under random node, route, and transfer-edge removal. (**d**–**f**) LWCC AUC under targeted strategies T1 (hyperdegree), T2 (betweenness), and T3 (transfer-first). Thin gray curves represent individual cities; thick green (random) and red (targeted) curves show the cross-city median, with shading indicating the interquartile range.

We first measure connectivity retention as nodes, routes, and transfer edges are removed. Transfer-first attack is most damaging in 33 of 45 cities (Figure 3; Table 3). Median LWCC AUC falls from 0.219 under random node removal to 0.124 under transfer-first attack. Betweenness and hyperdegree targeting give 0.148 and 0.156, respectively. The T3–T2 gap is significant (paired Wilcoxon $p=1.3\times 10^{-5}$; rank-biserial r=0.69). T2 is more damaging in 12 cities, often those with high metro-node ratios where central stations are not transfer points (Supplementary Figure S15). Random route and transfer-edge removal are milder. R3 has median LWCC AUC 0.964 because the bus layer remains connected after intermodal links disappear. Cross-mode LCC nevertheless collapses. Thus, within-mode connectivity and cross-mode integration can diverge.

### 3.3. Targeted Triggering Is Associated with Deeper Route-Support Pruning

**Figure 4:**
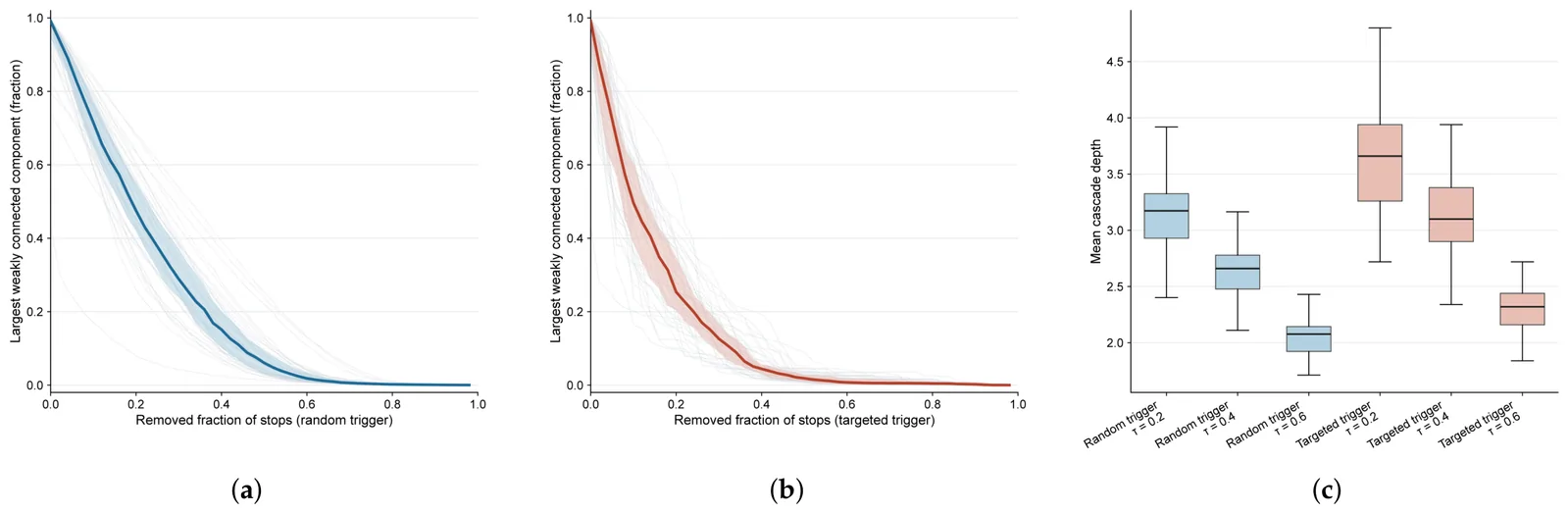
Cross-city cascade results. (**a**) LWCC under random-node triggering (τ=0.2, median and IQR). (**b**) LWCC under targeted-node triggering. (**c**) Mean cascade depth at τ∈{0.2,0.4,0.6}.

Static retention measures immediate fragmentation. We next test whether initial damage causes additional route-support pruning. Targeted damage produces deeper cascades than random damage in 41 of 45 cities (Figure 4; Table 3). At τ=0.2, median depth is 3.66 rounds under targeted triggering and 3.17 under random triggering (paired Wilcoxon $p<10^{-7}$; rank-biserial r=0.82). The contrast remains at τ=0.6. Under 10% random damage, few nodes cross the tolerance threshold. The main difference at this operating point is, therefore, depth, not universal secondary amplification. Broader pruning appears at τ≥0.4 or under targeted initial damage (C4 median LWCC AUC 0.148).

Transfer-edge removal shows why LWCC alone is insufficient. In the six-city exploratory R3 experiment, median LWCC AUC is 0.900. Median cross-mode LCC AUC is 0.858, and median cross-mode reachability AUC is 0.843 (Supplementary Table S19). Within-mode connectivity, therefore, remains relatively high while cross-modal function declines more strongly. LWCC is the primary general-connectivity endpoint across disruption protocols; for the R3 experiment, cross-mode LCC and cross-mode reachability are co-primary endpoints for multimodal integration.

### 3.4. Potential Proximity Coupling Shows Different Associations Across Resilience Diagnostics

The cross-city regressions show that potential proximity coupling is associated differently with the three outcome families. PPCR is the strongest single correlate of random-damage connectivity retention across cities (Pearson r=0.807, $R^{2}=0.652$, $p<10^{-11}$). Under random node removal, cities with higher inferred proximity coupling retain more connectivity, and the same positive association also appears under betweenness-targeted attack (T2, r=0.531, $p<10^{-3}$). When inferred proximity-coupled nodes are held immune from the random removal pool, the association strengthens further (r=0.906, Supplementary Material, Section S10). These results identify PPCR as a structural correlate, not a validated measure of passenger interchange, of static connectivity retention.

Route-support outcomes show a different pattern. PPCR is positively associated with cascade depth (r=0.589) and collapse breadth (r=0.680), indicating that systems with higher inferred proximity coupling tend to show deeper pruning under targeted triggering. However, PPCR is not the only correlate of route-support outcomes. Log-transformed route count is the strongest correlate of cascade depth (r=0.767, $R^{2}=0.588$, $p<10^{-9}$), whereas average route size is negatively associated with collapse breadth under targeted triggering (r=−0.747, $R^{2}=0.558$, $p<10^{-8}$). These associations show that retention and route-support pruning do not depend on exactly the same structural predictors.

**Figure 5:**
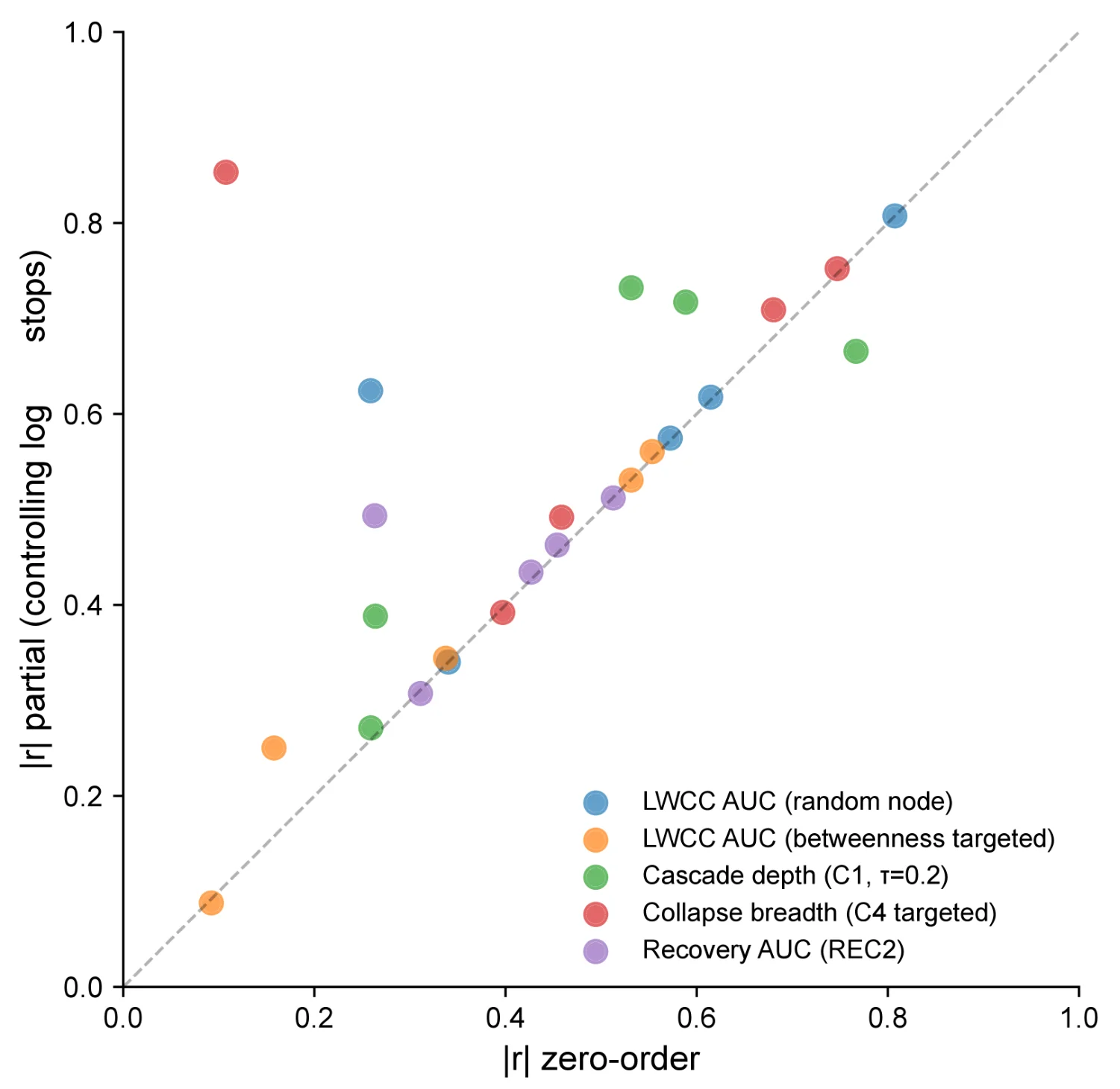
Partial versus zero-order correlations of each predictor with each outcome, controlling for $\log_{10}$ stops. The y-axis reports absolute correlation magnitude, |r|; filled circles identify PPCR.

**Figure 6:**
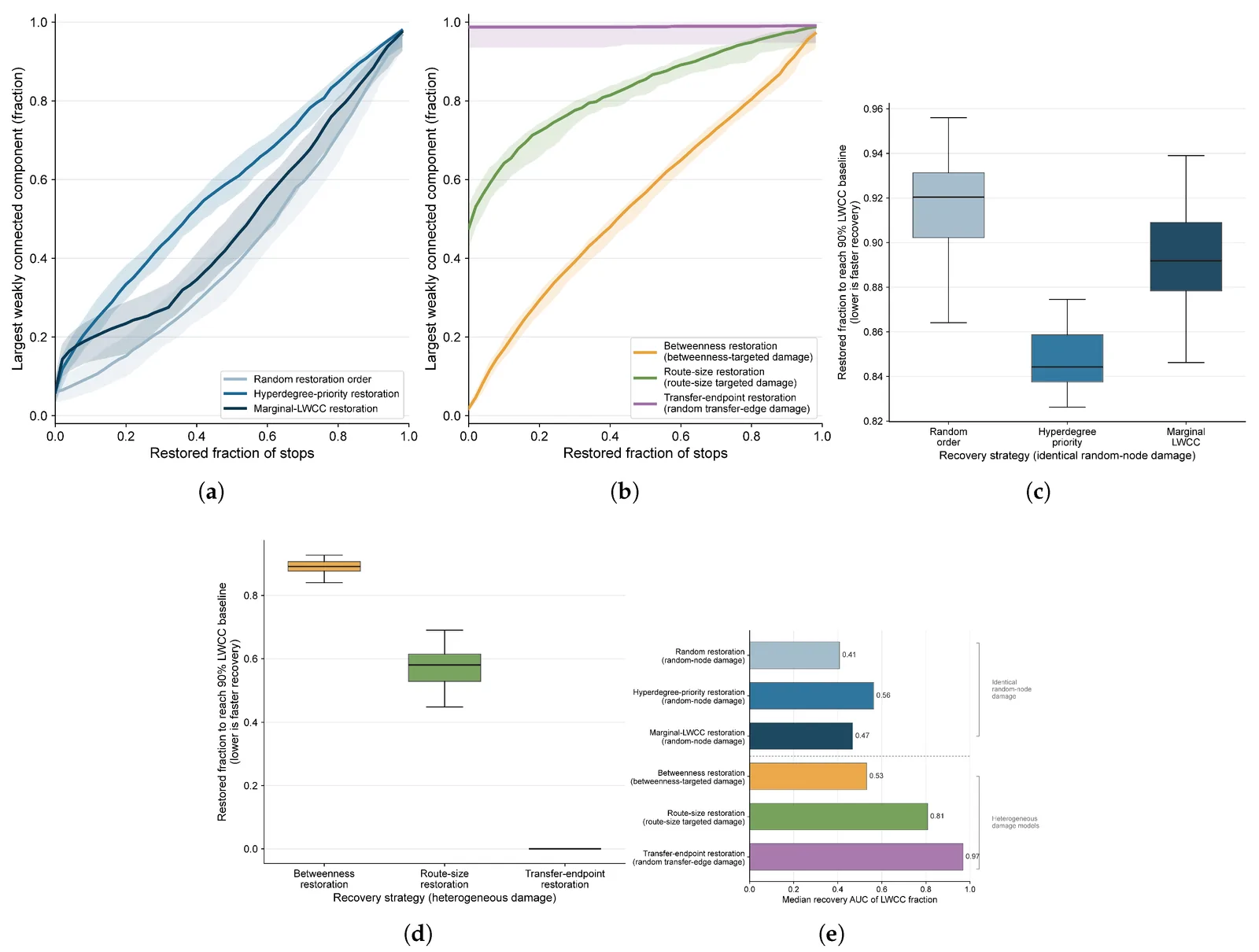
Cross-city recovery performance. (**a**) Recovery under matched random-node damage (REC1, REC2, REC6). (**b**) Recovery under heterogeneous damage (REC3–REC5). In (**a**,**b**), solid lines show cross-city medians and shaded bands indicate interquartile ranges (25th–75th percentiles). (**c**,**d**) $t_{90}$ across strategies. (**e**) Median recovery AUC.

Figure 5 summarizes zero-order and partial predictor–outcome correlations, with the latter adjusted for log stop count. Recovery AUC under hyperdegree-priority recovery is also positively associated with PPCR (r=0.513, $R^{2}=0.263$, p<0.001), but this relationship is weaker than those observed for connectivity retention and route-support outcomes and does not remain significant under joint adjustment (p=0.197 in the six-predictor model; Supplementary Material, Table S3). Under matched random-node damage, hyperdegree-priority recovery raises the median recovery AUC from 0.408 to 0.563 (paired Wilcoxon $p<10^{-8}$, paired Cohen’s d=3.40; Figure 6a), and the fraction of nodes needed to recover 90% of baseline performance ($t_{90}$) falls from 0.92 to 0.84 (Figure 6c). The marginal-LWCC heuristic (REC6) is intermediate (median 0.468; Table 3). These results indicate that recovery captures a related but weaker structural pattern.

**Figure 7:**
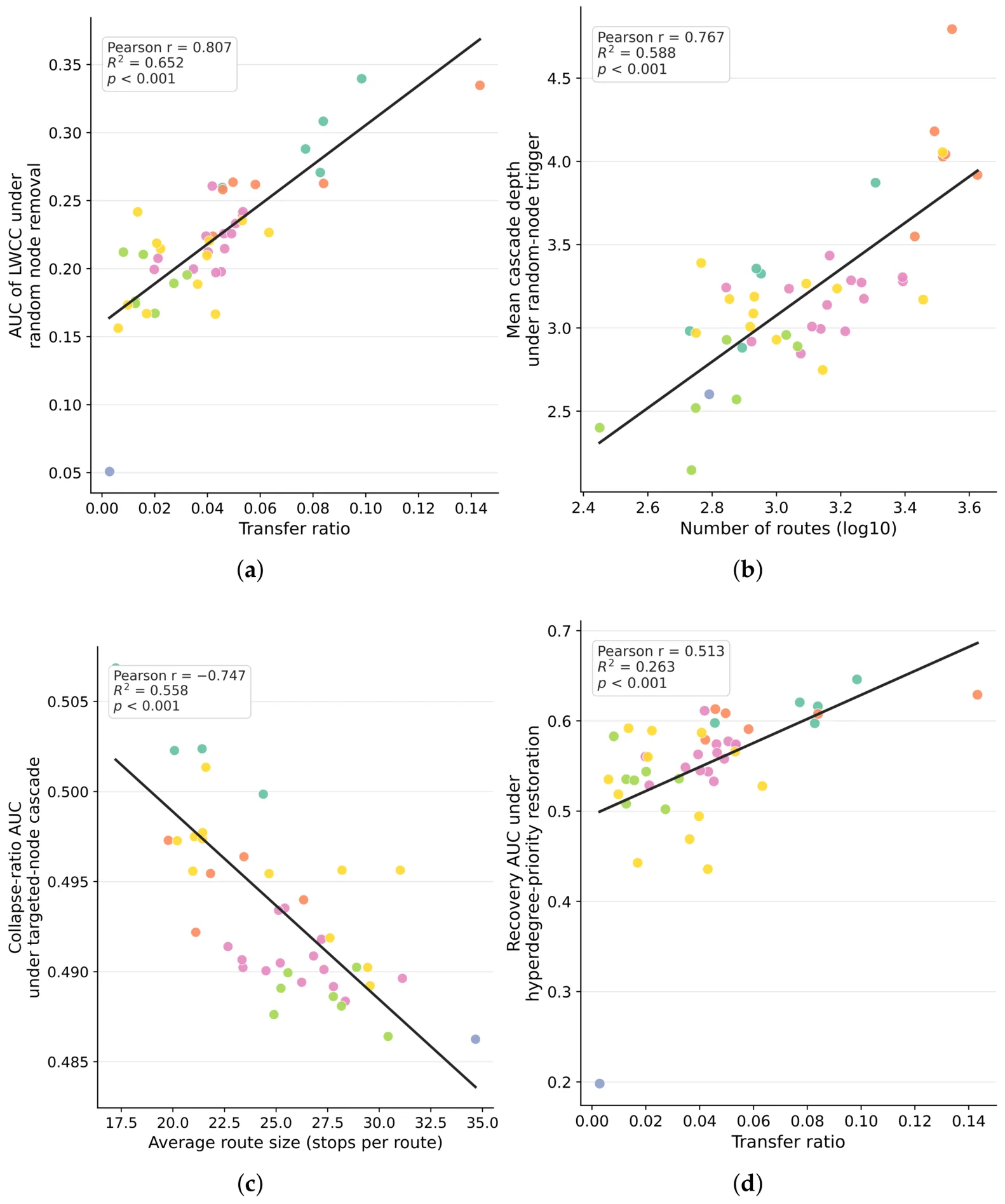
Structural correlates of resilience (n=45 cities). (**a**) PPCR vs. LWCC AUC under random node removal. (**b**) $\log_{10}$(routes) vs. cascade depth (C1). (**c**) Average route size vs. collapse breadth (C4, targeted). (**d**) PPCR vs. recovery AUC (REC2). Lines: OLS with 95% CI.

The five resilience measures are only moderately intercorrelated (mean |r|=0.52; Supplementary Table S12 and Figure S4). Three principal components are needed to explain 90% of their variance. The first explains 62.6%; the remainder is organized largely by route-support outcomes. No single ranking captures all five dimensions (Figure 7). The associations largely persist under joint adjustment. Because log stop count and log route count are nearly collinear (VIF >400), the reduced regressions use log stop count, PPCR, and metro-node ratio. All VIFs are below 2.5. LASSO resampling is compatible with this parsimonious specification, but predictor selection is not unique (Supplementary Tables S2 and S3). Controlling for size leaves the PPCR association with static retention unchanged (r=0.807) and raises the depth association from r=0.589 to 0.717. Leave-one-out $Q^{2}$ values are 0.54 for static retention, 0.72 for depth, 0.64 for breadth, and 0.05 for recovery AUC (Supplementary Table S4). Controlling for mean inter-stop distance also changes little (Supplementary Table S8). FDR correction retains the PPCR associations with static retention and cascade outcomes, but not recovery (Supplementary Table S6). These results confirm that no single diagnostic describes the full resilience profile.

The frozen structure-only replication set and the outcome-informed exploratory set are compared in Table 4.

### 3.5. Cross-Mode Connectivity as a Descriptive Interpretation of PPCR

The preceding analyses identify PPCR as a clear structural correlate of several outcomes. We clarify one topological aspect using cross-mode LCC. This is the largest connected component containing both bus and metro nodes, expressed as a fraction of all nodes.

**Figure 8:**
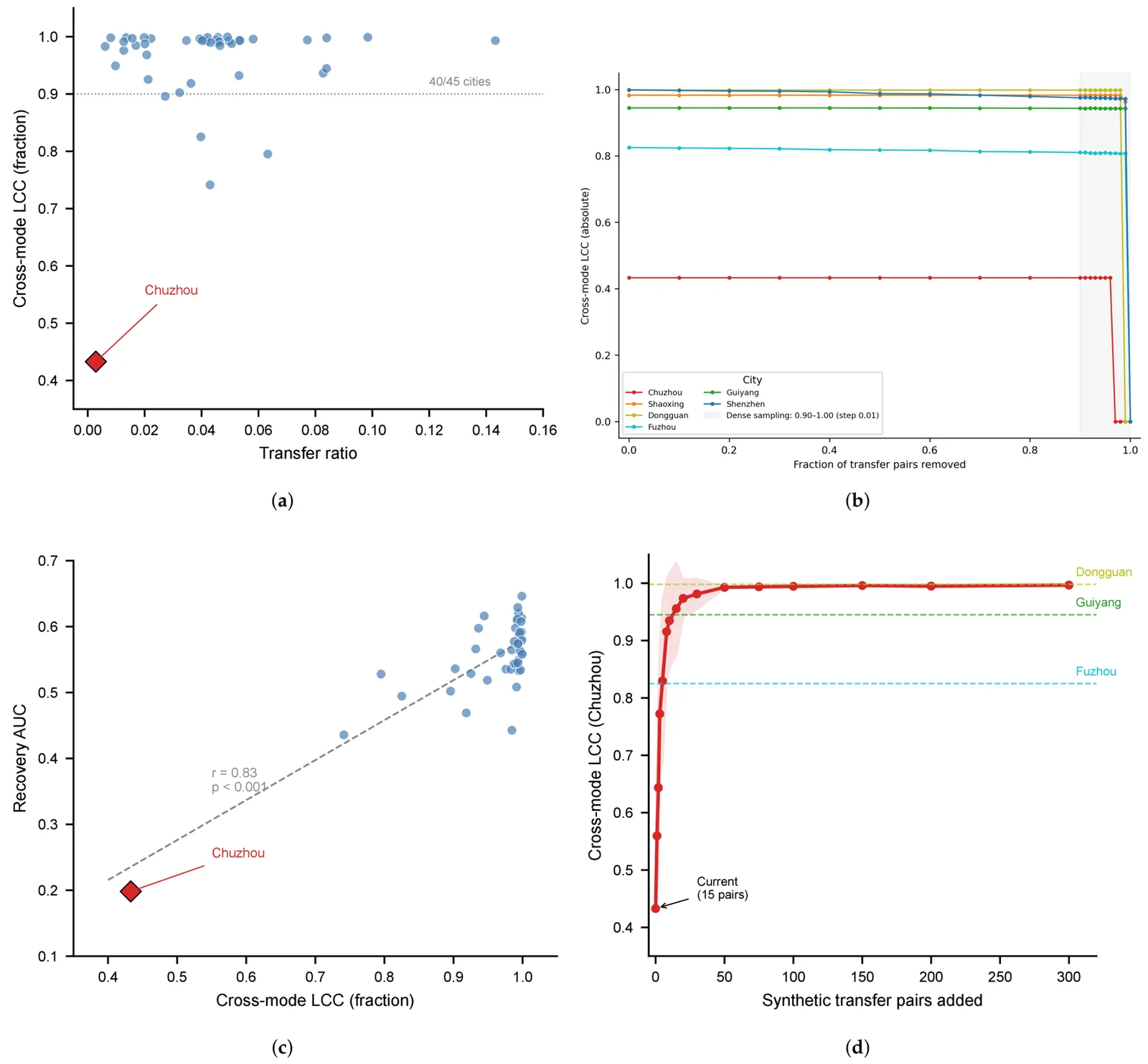
Cross-mode connectivity and a descriptive coupling threshold. In (**a**,**c**), blue circles represent the other 44 cities, and the red diamond marks Chuzhou. (**a**) Cross-mode LCC versus PPCR (45 cities). (**b**) Cross-mode LCC under progressive transfer-pair removal; the 0.90–1.00 interval is sampled at 0.01 increments and the markers are the evaluated removal fractions. Connecting segments are visual guides only. (**c**) Cross-mode LCC versus recovery AUC. (**d**) Cross-mode LCC for Chuzhou versus number of added synthetic transfer pairs.

Across the 45-city sample, 40 cities have cross-mode LCC above 0.90, indicating near-complete structural integration of bus and metro (Figure 8a). Chuzhou is a pronounced outlier: its cross-mode LCC is 0.433, meaning that fewer than half of its nodes belong to a component shared by both modes. The next-lowest city (Dalian) has cross-mode LCC of 0.741. Chuzhou’s 15 transfer pairs, connecting 5273 bus nodes to 17 metro nodes, are insufficient to bridge the two subnetworks into a single component. The median across the sample is 257 transfer pairs (Supplementary Material, Table S1).

As an exploratory illustration, we progressively remove transfer pairs from six cities (Figure 8b). In Shenzhen and Guiyang, cross-mode LCC remains above 0.97 and 0.94, respectively, after 90% removal. We sample the final interval from 90% to 100% in 1% increments. Shenzhen remains at 0.972 and Guiyang at 0.943 after 99% removal; both reach zero at 100% (Supplementary Table S27). Chuzhou reaches zero at 97%, whereas Dongguan reaches zero at 99%. The endpoint transition is, therefore, discrete and city specific. Dense systems retain cross-mode connectivity until their final links disappear, while Chuzhou is weakly integrated at baseline.

Cross-mode LCC is also positively associated with recovery AUC across the 45-city sample (r=0.834, $p<10^{-12}$; Figure 8c). Excluding Chuzhou lowers the Pearson correlation to r=0.568, while the rank correlation changes only slightly (Spearman ρ=0.570→0.540; Supplementary Material, Table S5). This pattern indicates that cross-mode LCC separates weakly integrated systems from highly integrated ones more clearly than it orders the dense group internally.

A counterfactual experiment on Chuzhou further illustrates the same point (Figure 8d). Adding 10 randomly placed synthetic transfer pairs raises cross-mode LCC from 0.433 to 0.935 (±0.082 across 20 trials), saturating near 0.99 at approximately 50 additional pairs. These results show how sparse or abundant transfer links alter cross-mode integration in the projected network.

### 3.6. Robustness and Sensitivity Analysis

We next test sensitivity to the walk-transfer definition and the number of Monte Carlo repetitions. Unless stated otherwise, the network-version, threshold, convergence, and model-comparison displays use the six outcome-informed exploratory cities. Median relative drift across four network versions is 2.37% for random disruption, 4.13% for targeted disruption, 4.93% for route-support metrics, and 1.58% for recovery. Static-retention and recovery rankings are stable, whereas route-support rankings are less stable. The factorial model comparison is replicated on the frozen structure-only set and reported below as the confirmatory check.

We compare three representations under identical attack seeds: a P-space clique expansion, a sequential L-space projection, and the full hypergraph. P-space and L-space are descriptive shorthands for clique-based route projection and sequential stop-to-stop projection. Under static targeted attack, P-space yields an LWCC AUC about 1.7–2.6 times that of L-space because the induced cliques add redundancy.

We then apply the same route-support rule to both pairwise projections after externally restoring route membership. At 10% random damage and τ=0.30, all three implementations give identical outcomes in every city and repetition. Mean collapse is 0.1001±0.0002. Without restored membership, the neighbour-fraction rule on L-space gives mean collapse 0.9877±0.0033. The rule, therefore, governs the numerical outcome. The hypergraph’s contribution is to preserve route membership natively instead of requiring external reconstruction.

The additional P-space reconstruction check is summarized in Table 5. It establishes what can and cannot be reproduced without the hypergraph: the route-support cascade can be reproduced numerically when the original route-incidence table is supplied externally, whereas a pairwise graph alone cannot define route viability or route-level support loss.

**Figure 9:**
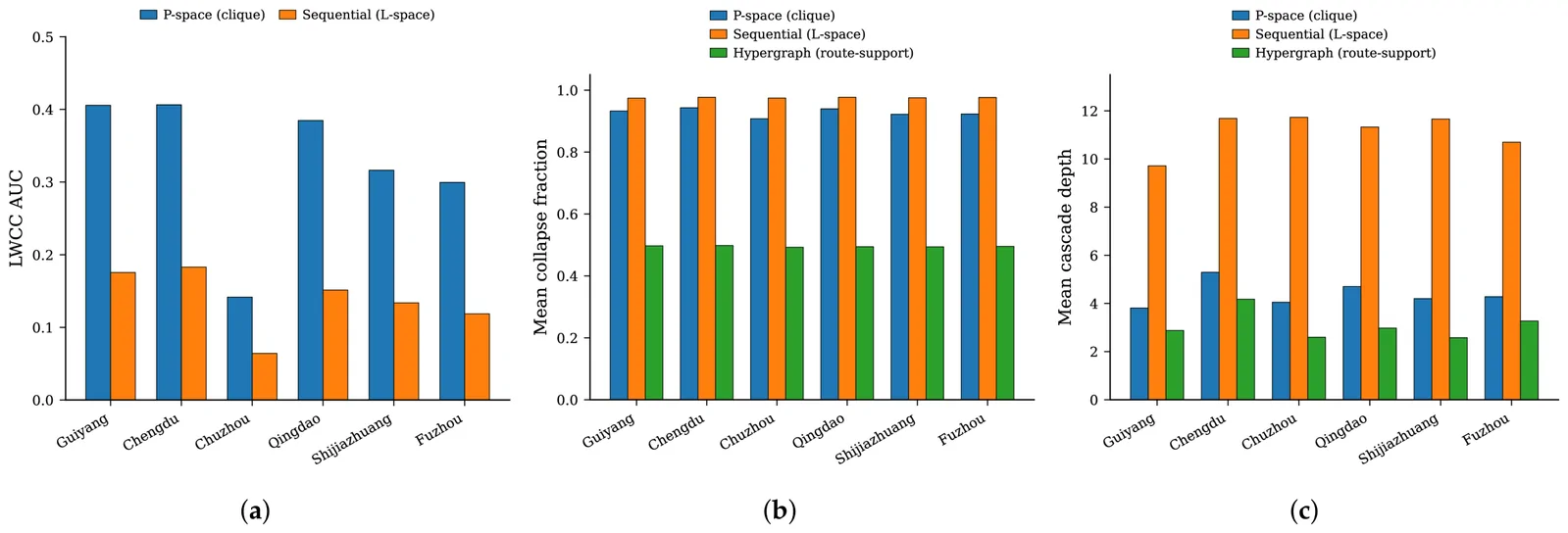
Exploratory cascade comparison across three representations (C1, τ=0.3, six outcome-informed cities; means in Table 6). (**a**) LWCC AUC. (**b**) Collapse fraction. (**c**) Cascade depth.

Under the neighbour-fraction cascade rule (τ=0.3), both graph projections collapse almost completely (mean collapse fractions 0.922–0.977). Under the route-support rule on the hypergraph, the same six exploratory cities remain partially intact (collapse fraction 0.493–0.498) and show shallower cascade depths (Table 6; Figure 9). The sequential (L-space) projection shows nearly the same collapse level (0.976) as the clique expansion (0.928). In this outcome-informed exploratory set, changing the cascade rule produces a larger difference in outcomes than changing the underlying representation alone.

**Figure 10:**
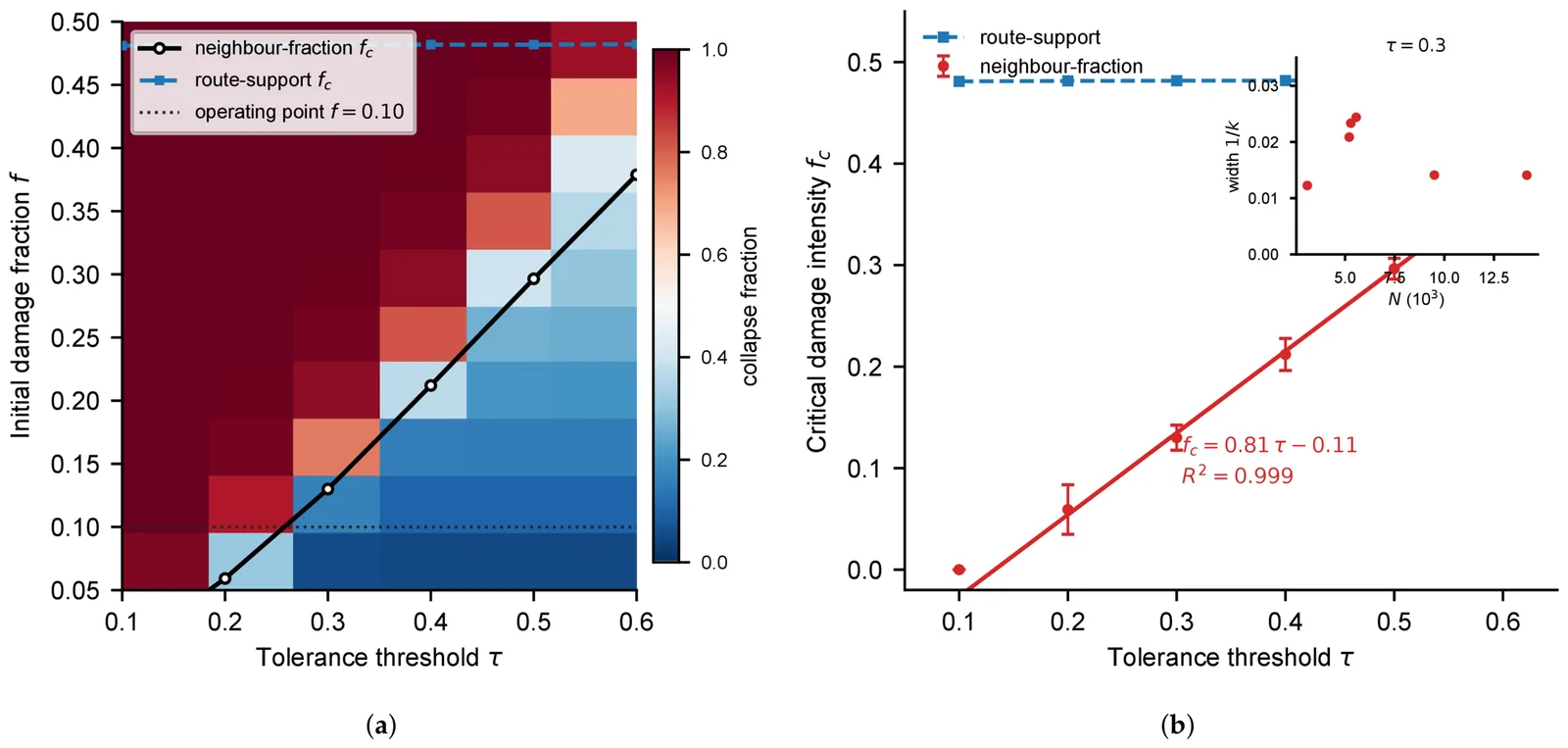
Exploratory cascade phase diagram on the P-space clique projection (six outcome-informed cities). (**a**) Neighbour-fraction collapse fraction in the (f,τ) plane; curves: logistic midpoint $f_{c}\left(\tau \right)$ for neighbour-fraction (black) and route-support (blue dashed) rules; dotted line: f=0.10. (**b**) Fitted $f_{c}$ versus τ; the fitted equation is placed in an unoccupied lower-right region and the inset is separated from the data to preserve readability. Inset: transition width 1/k at τ=0.3 versus *N*.

Although both rules use τ=0.3, the threshold refers to different quantities: surviving routes in the route-support rule and surviving neighbours in the neighbour-fraction rule. The two rules, therefore, generate different damage–tolerance maps on the same underlying systems (Figure 10). For the neighbour-fraction rule, the fitted midpoint $f_{c}\left(\tau \right)$ scales linearly with tolerance, $f_{c}\approx 0.81\;\tau -0.11$ ($R^{2}=0.999$). For the route-support rule, the fitted midpoint remains near 0.50 across the tested tolerance range (mean ${\bar{f}}_{c}=0.498\pm 0.002$ across the six outcome-informed exploratory cities and three tolerance levels τ∈{0.2,0.4,0.6}; per-city values in Supplementary Material, Table S15). Across these exploratory cities, the transition width remains narrow (<0.025 at τ=0.3; Figure 10b inset), indicating a threshold-like crossover rather than a broad gradual transition.

**Figure 11:**
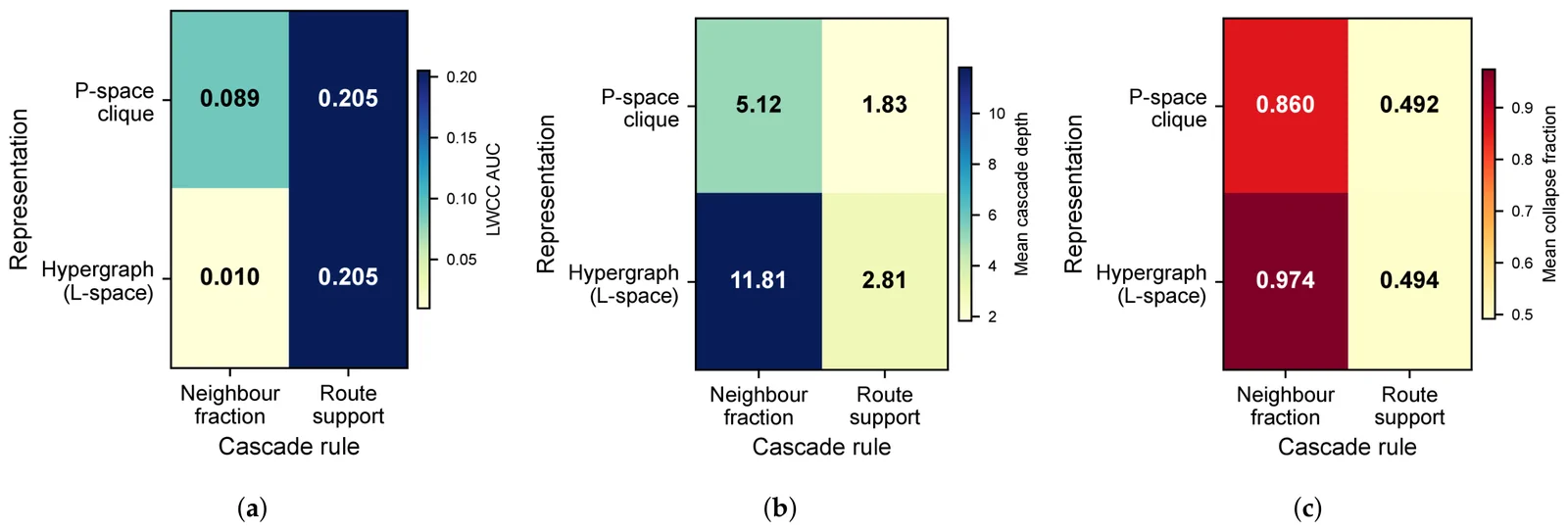
Exploratory factorial decomposition of cascade outcomes (representation × cascade rule, six outcome-informed cities). Each cell is the mean over the six cities. (**a**) LWCC AUC. (**b**) Mean cascade depth. (**c**) Mean collapse fraction.

A 2×2 factorial analysis quantifies the contrast (Table 7; Figure 11). In the outcome-informed exploratory set, switching from neighbour-fraction to route-support raises mean LWCC AUC by 0.156, reduces depth by 6.14 rounds, and reduces collapse fraction by 0.424. Representation has smaller effects on AUC (0.040) and collapse (0.058). More importantly, the frozen structure-only set (Guangzhou, Shanghai, Tianjin, Xuzhou, Zhengzhou, and Urumqi) gives a rule effect on collapse of 0.429, within 1.2% of the exploratory estimate; the representation effect remains approximately 0.05. This structure-only replication is the confirmatory city-set comparison (Table 4; Supplementary Sections S4 and S25). In the damage sweep, neighbour-fraction on P-space enters a high-collapse regime near f=0.13–0.25. Route-support remains in a low-damage regime over the same interval (Supplementary Section S11).

Table 8 compares the three cascade models under matched initial conditions.

**Figure 12:**
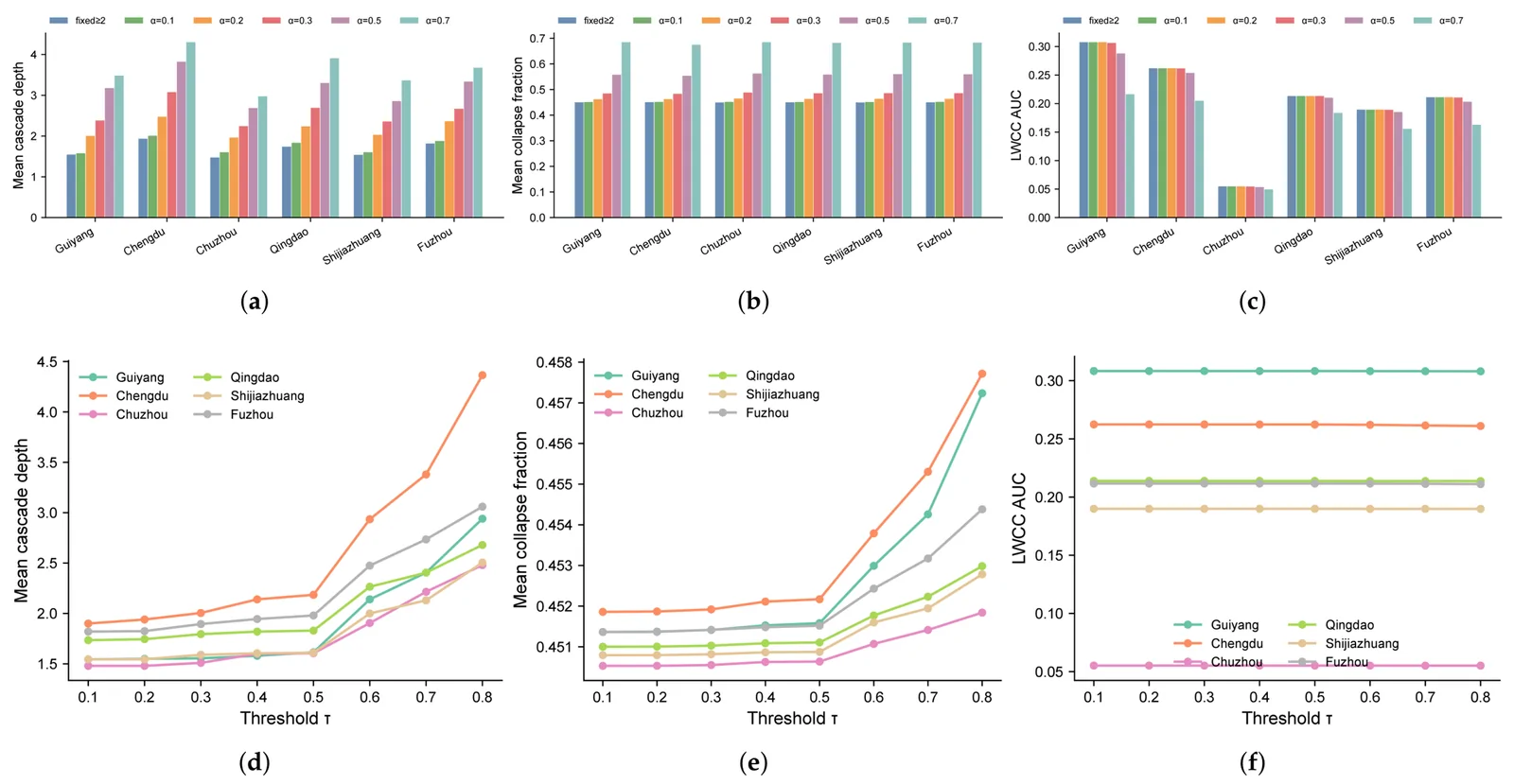
Cascade sensitivity analysis. (**a**–**c**) Sensitivity to route-viability condition (α∈{0.1,0.2,0.3,0.5,0.7}) at τ=0.2. (**d**–**f**) Sensitivity to τ (0.1–0.8) at fixed min-viable ≥2.

In the six outcome-informed exploratory cities, route viability changes the outcomes more than τ at the tested operating point. Proportional thresholds increase depth and collapse breadth and lower LWCC AUC in all six cities (Figure 12a–c). Mean collapse rises from 0.451 under the two-stop condition to 0.683 at α=0.7. Mean depth rises from 1.68 to 3.63, and LWCC AUC falls from 0.207 to 0.163. By contrast, changing τ from 0.1 to 0.8 changes mean collapse only from 0.451 to 0.454, although depth rises from 1.67 to 3.01 (Figure 12d–f). Supplementary Tables S16 and S17 report city-level estimates and uncertainty intervals.

For transport interpretation, the practical implication is not that one metric should replace all others, but that multimodal resilience assessment benefits from combining connectivity-based, route-dependent, and recovery-based diagnostics. In particular, high-PPCR systems may look comparatively robust under one diagnostic while appearing more dependent under another.

The main values are stable across network versions and Monte Carlo settings, but ranking stability differs by metric. Static-retention and recovery rankings are highly stable (mean ρ>0.97). Cascade-depth rankings are more sensitive to the transfer definition. Within each network version, targeted triggering nevertheless produces deeper pruning than random triggering. The main structural correlates also remain stable across the tested thresholds. Relative to the 200 m reference, PPCR rankings are highly concordant at 100 m (ρ=0.974) and 300 m (ρ=0.996). The name-match-only definition is less concordant (ρ=0.428; Supplementary Section S13 and Table S13). This tests robustness of the proxy; it does not validate realized transfers.

The relative-drift estimates and cross-version ranking stability are summarized in Table 9 and Table 10, respectively.

**Figure 13:**
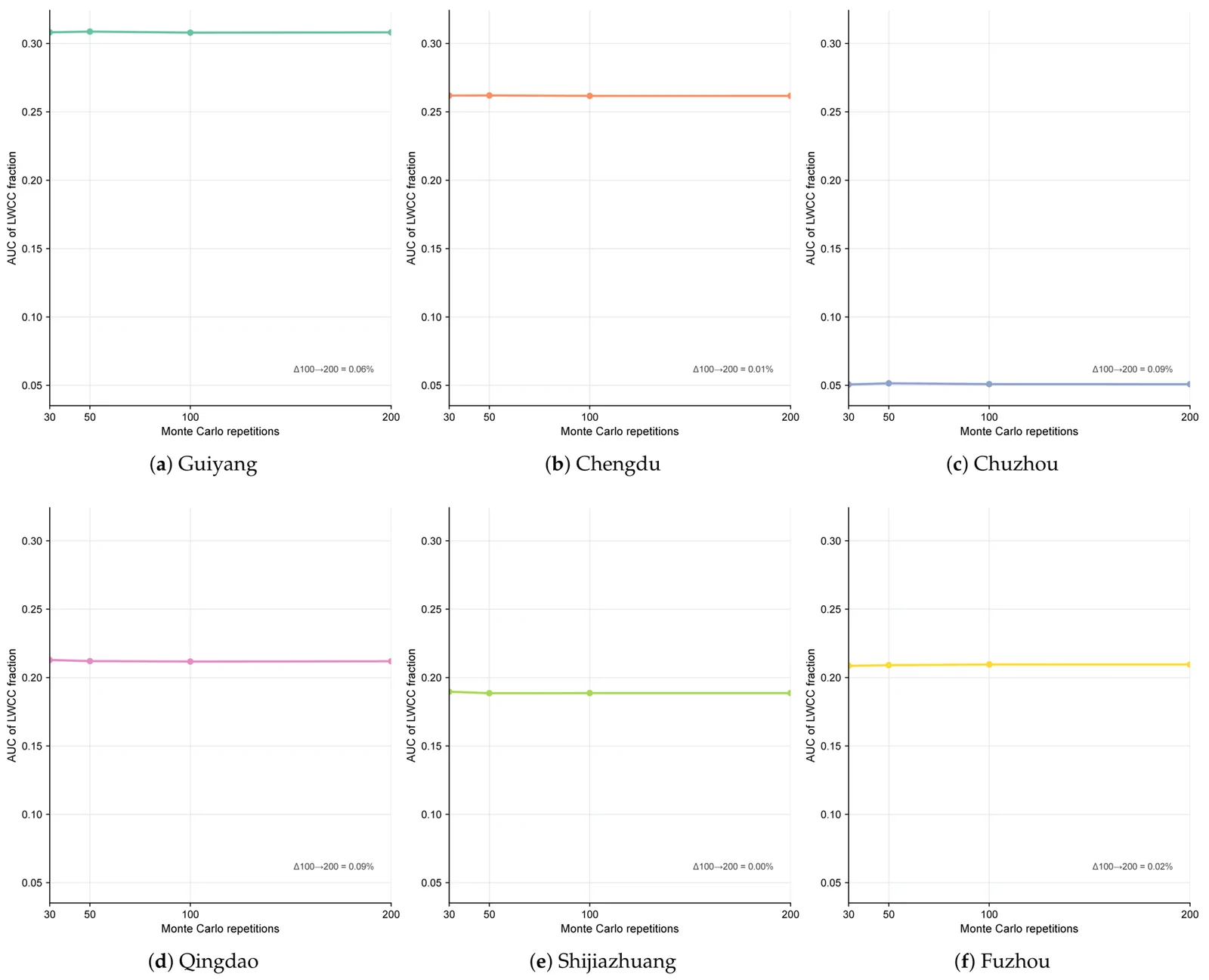
Monte Carlo convergence across the six outcome-informed exploratory cities.

One hundred repetitions give negligible deviation from the 200-repetition reference (Figure 13).

## 4. Discussion

PPCR is associated with multimodal resilience only under specific diagnostics. Targeted disruption is more damaging than random removal, and structurally important nodes remain critical. Our framework changes the interpretation of this criticality. In static tests, high-PPCR cities retain more of their LWCC, consistent with redundant paths between modes. Under targeted triggering, the same cities tend to show deeper route-support pruning. The same inferred coupling can, therefore, align with different outcomes depending on the failure regime and metric.

Previous multimodal studies show that coupling can support connectivity while increasing vulnerability. They usually represent coupling through pairwise links or layer dependencies. Our results agree that targeted disruption matters more than random damage. They also show that route-level dependence changes how vulnerability is measured. Static retention, route-support pruning, and recovery have different structural correlates. The first principal component explains 62.6% of the five-outcome variance, leaving substantial outcome-specific structure. Structural-only and outcome-informed clustering also produce different partitions (Supplementary Section S4). In Chuzhou, adding 10 synthetic transfer pairs raises cross-mode LCC from 0.43 to 0.93.

The broader implication is that multimodal integration is associated with different forms of vulnerability. Added connectivity can provide redundancy, but it can also concentrate route overlap at transfer locations. A transfer node is, therefore, not important in a context-free sense. Its importance depends on whether the metric emphasizes paths, route viability, or restoration. The practical task is to identify which dependencies matter under each disruption. Our main contribution is this mismatch across diagnostics.

The hypergraph does not produce a unique numerical cascade when route membership is restored externally to a graph. Its contribution is representational: route–node incidence remains native, transparent, and directly queryable. Directed projections remain suitable for path metrics, but a pairwise adjacency matrix alone cannot evaluate route viability or route-support loss. The factorial analysis confirms that the propagation rule contributes more to the outcome gap than representation alone. Because city rankings vary across transfer definitions, route-support depth is best interpreted as a group-level diagnostic rather than a league table.

Because these data are cross-sectional, statements that integration “redistributes vulnerability” or “creates new dependencies” are interpreted here as descriptive summaries of the observed association pattern under the tested counterfactual scenarios, not as causal claims.

Changing the cascade rule affects outcomes more than changing representation alone. This does not establish numerical superiority of the hypergraph. Rather, route-support requires route membership as a first-class object. When that membership is restored to L-space, the result matches the native hypergraph. The near-total collapse under neighbour-fraction arises from a different rule applied without route context. Representation and rule are, therefore, not fully independent in practice. The hypergraph makes route-dependent survival native instead of requiring deliberate reconstruction.

The route-support midpoint $f_{c}$ remains near 0.50 across the tested tolerance range, whereas the neighbour-fraction midpoint scales with τ. Equation (14) provides a qualitative explanation. A node with hyperdegree $d_{H}\left(v\right)$ fails after losing at least $k\left(v\right)=\lceil \tau d_{H}\left(v\right)\rceil$ routes. Under random damage, the route-loss distribution becomes more concentrated as hyperdegree grows. The high-overlap core then anchors the system-level transition. This interpretation is qualitative; an analytic derivation for heterogeneous hypergraph *k*-core processes remains future work. Empirically, diffuse random damage rarely crosses the route-support threshold, while targeted concentration is more consequential.

Several limitations define the scope of the conclusions. First, the analysis is structural rather than operational. We do not observe passenger volumes, service frequencies, capacity, timetable coordination, or rerouting. The metrics, therefore, describe structural potential, not realized passenger impact. PPCR is inferred from names and a 200 m distance rule; it is not observed transfer demand. The cascade rule is also a stylized abstraction rather than a calibrated failure model. Alternative route-viability conditions materially change cascade magnitude.

Second, the cascade results are relative vulnerabilities under controlled damage. They are not estimates of passenger loss, interruption duration, or real-world failure probability. Recovery is likewise a structural benchmark. The heuristic assumes complete knowledge of the damage state, and its adjusted cross-city association is weak. Recovery AUC has a leave-one-out $Q^{2}$ of 0.05, so the three aggregate predictors have little out-of-sample predictive value for recovery. City-specific spatial configurations probably matter more than these aggregate descriptors.

Third, the sample is a cross-section of 45 Chinese cities. Residual regional dependence cannot be excluded. We, therefore, report FDR checks, leave-one-city-out analyses, and explicit exclusion of Chuzhou. Chuzhou remains an important low-coupling boundary case. Finally, several expensive sensitivity displays use six outcome-informed cities and are explicitly exploratory. The main cross-city results use all 45 cities, and the factorial comparison is replicated on the frozen structure-only set.

PPCR is a proxy for potential spatial coupling, not realized passenger transfers. The inferred links have not been validated against station maps, GTFS/NeTEx interchange fields, smart-card data, or observed walking paths. Threshold sensitivity tests alternative geometric definitions; it does not provide behavioural validation.

The statistical conclusions are limited to internal cross-sectional stability. No independent city set or later data release was available. We, therefore, report bootstrap intervals, model-selection frequencies, leave-one-city-out checks, and FDR sensitivity. We do not claim causal effects or replication in new cities.

Future work should, therefore, move from structural comparison toward operational validation. The present CPTOND-2025 release contains structural route, stop, mode and coordinate fields but no passenger volumes, service frequencies, vehicle capacities, timetable coordination, observed transfer paths, or disruption and recovery records. These missing operational data prevent calibration of the transfer rule, the route-support threshold, and passenger-impact endpoints in the present study. The most direct extensions are to link the structural hypergraphs to GTFS/NeTEx interchange fields, smart-card or observed walking transfers, service-frequency and capacity records, passenger-flow data, and dated disruption/recovery logs. This extension will allow city-specific transfer validation, demand-weighted accessibility, affected-passenger estimates, and empirical calibration of τ and route viability before operational claims are made. It should also test whether the route-support mechanism remains informative under spatially correlated failures and richer behavioural assumptions. More broadly, multimodal integration should be judged not by a single robustness score, but by the distinct forms of dependence it creates and the failure regimes under which those dependencies become consequential.

## 5. Conclusions

We developed a route-preserving bus–metro hypergraph in which node viability depends on surviving routes rather than pairwise adjacency alone. Across 45 cities, transfer-first attack is the most damaging static strategy in most cases. Connectivity retention, route-support pruning, and recovery are only moderately aligned. Multimodal resilience, therefore, cannot be summarized by one robustness ranking.

Multimodal integration should be evaluated through both added connectivity and route dependence. PPCR does not have one meaning across diagnostics: it aligns with stronger retention in one regime and deeper pruning in another. Structural-only and outcome-informed clustering also produce different partitions. Resilience information is, therefore, not a restatement of basic structure. The higher-order formulation makes route-dependent structural failure directly observable in the model.

## Figures and Tables

**Table 1:** Disruption, cascade, and recovery scenarios used in the study.

Family	Code	Protocol	MC Reps
Random disruption	R1	All-mode node removal (fraction 0–1 in steps of 0.02)	100
	R2	All-mode route removal	100
	R3	Transfer-edge removal	100
Targeted disruption	T1	Deterministic node removal ordered by hyperdegree	†
	T2	Deterministic node removal ordered by betweenness	†
	T3	Deterministic transfer-node-first removal	†
	T4	Deterministic route removal ordered by route size	†
	T5	Deterministic route removal ordered by route betweenness	†
	T6	Deterministic node removal ordered by sum of hyperdegrees at transfer endpoints	†
Cascade (τ: route-support tolerance)	C1–C3	Route-support cascade; random node triggering at τ∈{0.2,0.4,0.6}	100
	C4	Route-support cascade; targeted node triggering (τ=0.2)	†
	C5	Route-support cascade; random transfer-edge triggering (τ=0.2)	100
Recovery (50% initial damage)	REC1	Random recovery order; random damage (baseline)	100
	REC2	Hyperdegree-priority recovery; random damage	100
	REC3	Hyperdegree-priority recovery; hyperdegree-targeted damage	100
	REC4	Hyperdegree-priority recovery; betweenness-targeted damage	100
	REC5	Hyperdegree-priority recovery; transfer-targeted damage	100
	REC6	One-shot marginal-LWCC greedy recovery; random damage	100

† Deterministic protocol; Monte Carlo repetitions not applicable. LWCC: largest weakly connected component. MC reps: Monte Carlo repetitions per attack-fraction level.

**Table 2:** Outcome-informed descriptive grouping of the 45-city sample and each cluster’s exploratory example. Clusters are denoted as Cluster 1–6 (or K1–K6) to distinguish them from cascade scenarios C1–C5.

Cluster (K)	Representative	*n*	Structural Type	Med. $\log_{10}$ (Stops)	Med. $\log_{10}$ (Routes)	Med. PPCR
1	Guiyang	5	Compact high-PPCR	3.491	2.938	0.083
2	Chengdu	6	Large hub-dominant	4.170	3.522	0.054
3	Chuzhou	1	Minimal-metro outlier	3.723	2.791	0.003
4	Qingdao	14	Mid-size baseline	3.934	3.161	0.044
5	Shijiazhuang	7	Long-route sparse	3.717	2.845	0.016
6	Fuzhou	12	Low-transfer vulnerable	3.715	2.965	0.029

**Table 3:** Cross-city benchmarks for disruption, cascade, and recovery (n=45 cities).

Scenario	Metric	Median	Q1	Q3	Min	Max
R1	LWCC AUC	0.219	0.197	0.242	0.051	0.340
R2	LWCC AUC	0.691	0.660	0.724	0.268	0.778
R3	LWCC AUC	0.964	0.918	0.973	0.424	0.979
T1	LWCC AUC	0.156	0.131	0.183	0.051	0.283
T2	LWCC AUC	0.148	0.121	0.164	0.064	0.201
T3	LWCC AUC	0.124	0.107	0.146	0.047	0.219
C1	LWCC AUC	0.219	0.197	0.242	0.051	0.338
C1	Depth	3.17	2.93	3.33	2.15	4.79
C4	LWCC AUC	0.148	0.121	0.163	0.064	0.200
C4	Depth	3.66	3.26	3.94	2.72	5.54
C5	Depth	3.12	2.96	3.35	2.82	4.20
REC1	Recovery AUC	0.408	0.368	0.453	0.088	0.597
REC2	Recovery AUC	0.563	0.534	0.591	0.198	0.646
REC6	Recovery AUC	0.468	0.428	0.520	0.114	0.641

Scenario codes are defined in Section 2.3; all cascades use τ=0.2. R2 = random route removal; R3 = random transfer-edge removal. REC3–REC5 pair hyperdegree-priority recovery with hyperdegree-, betweenness-, and transfer-targeted damage. R3 yields a high median LWCC AUC (0.964) because the bus layer alone remains largely connected once transfer edges are severed; cross-mode structural integration collapses while within-mode bus connectivity is preserved.

**Table 4:** Comparison of the frozen structure-only replication set and the outcome-informed exploratory set.

Metric	Value	Interpretation
Adjusted Rand Index	0.249	Low agreement
Representative overlap	0/6	No shared representatives
Rule effect on collapse (13-feature set)	0.424	
Rule effect on collapse (6-feature set)	0.429	Similar pattern

**Table 5:** Matched route-support reconstruction check (six outcome-informed exploratory cities, 10% initial random node damage, τ=0.30, 50 repetitions).

Representation and Rule	Mean Collapse Fraction	Mean Cascade Depth
Native hypergraph + route-support	0.1001±0.0002	identical to reconstructed versions
L-space + external route membership + route-support	0.1001±0.0002	identical to native hypergraph
P-space + external route membership + route-support	0.1001±0.0002	identical to native hypergraph
L-space + neighbour-fraction (no membership)	0.9877±0.0033	22–30 rounds

**Table 6:** Exploratory mean cascade outcomes across three representations (C1, τ=0.3, six outcome-informed cities; averaged over f∈[0,1]).

Representation	LWCC AUC	Mean Depth	Mean Collapse
P-space clique	0.047	4.39	0.928
Sequential (L-space)	0.009	11.14	0.976
Hypergraph (route-support)	0.205	3.08	0.495

**Table 7:** Exploratory factorial decomposition of cascade outcomes (2×2: representation × cascade rule, six outcome-informed cities).

Source	Δ LWCC AUC	Δ Depth	Δ Collapse
Grand mean	0.127	5.39	0.705
Main effect: representation	0.040	−3.83	−0.058
Main effect: cascade rule	−0.156	6.14	0.424
Interaction	0.079	−5.71	−0.112

**Table 8:** Exploratory cascade model comparison on six outcome-informed cities (10% random node removal, τ=0.3, 50 repetitions; neighbour-fraction rule on L-space projection).

	Route-Support		Neighbour-Fraction		Interdependent	
City	Collapse	Depth	Collapse	Depth	Collapse	Depth
Guiyang	0.100	1.2	0.991	29.3	0.337	4.2
Chengdu	0.101	2.7	0.987	37.9	0.396	4.9
Chuzhou	0.100	1.3	0.988	42.1	0.845	2.9
Qingdao	0.100	1.5	0.994	41.1	0.484	4.4
Shijiazhuang	0.100	1.1	0.985	38.0	0.545	4.0
Fuzhou	0.100	1.7	0.986	36.5	0.608	4.2
Mean	0.100	1.6	0.988	37.5	0.536	4.1

All three models use τ=0.3 under identical initial conditions; the neighbour-fraction rule here uses the sequential (L-space) projection. Route-support collapse equals the initial damage (0.100; zero secondary amplification), neighbour-fraction exceeds 0.98 (near-total failure) on this sparse projection, and the interdependent model is intermediate. On the denser P-space clique projection the same operating point stays in the low-damage regime (collapse ≈0.12; Figure 10). Route-support results are identical at $\tau_{\mathrm{RS}}=0.2$ (Supplementary Material, Table S7). Collapse: fraction of nodes failed at convergence; Depth: mean cascade rounds.

**Table 9:** Relative drift across four walk-transfer network versions.

Phase	Metric	Median Drift	IQR	Max Drift
Random disruption	LWCC AUC	0.0237	0.0363	0.1681
Targeted disruption	LWCC AUC	0.0413	0.1009	0.3175
Cascade failure	Cascade depth	0.0493	0.2344	0.4474
Recovery	Recovery AUC	0.0158	0.0282	0.0937

**Table 10:** Cross-version city-ranking stability (Spearman ρ).

Phase	Metric	Mean ρ	Min ρ	Max ρ
Random disruption	LWCC AUC	0.9714	0.9429	1.0000
Targeted disruption	LWCC AUC	0.9841	0.9429	1.0000
Cascade failure	Cascade depth	0.8089	−0.0857	1.0000
Recovery	Recovery AUC	0.9810	0.8286	1.0000

## Data Availability

The multimodal transit data used in this study were extracted from CPTOND-2025 (China Public Transport Operation Network Dataset, 2025 edition), which provides route-level and stop-level vector records for urban bus and metro systems as of June 2025. The dataset is publicly available at https://doi.org/10.6084/m9.figshare.29377427 under a CC-BY 4.0 license. The public reproducibility repository for this study is available at https://github.com/hostnametian/Hypergraph (accessed on 30 August 2026). The raw CPTOND-2025 files are obtained from the cited public source; their release identifier and preprocessing rule are recorded in the repository. The code and reproducibility metadata will remain openly available after publication.

## Supplementary Materials

The following supporting information can be downloaded at https://www.mdpi.com/article/10.3390/e28090976/s1, Supplementary File S1: *Supplementary Materials: Contrasting Resilience Diagnostics in Route-Preserving Multimodal Transit Hypergraphs* (Figures S1–S11 and Tables S1–S28).

## Abbreviations

The following abbreviations are used in this manuscript:

LWCC	Largest Weakly Connected Component
LSCC	Largest Strongly Connected Component
AUC	Area Under the Curve
LCC	Largest Connected Component
P-space	Clique-based route projection shorthand
L-space	Sequential stop-to-stop projection shorthand
IQR	Interquartile Range
PCA	Principal Component Analysis
OLS	Ordinary Least Squares
VIF	Variance Inflation Factor
LASSO	Least Absolute Shrinkage and Selection Operator
LOO	Leave-One-Out
CPTOND	China Public Transport Operation Network Dataset
