# Supplementary material for: Contrasting Resilience Diagnostics in Route-Preserving Multimodal Transit Hypergraphs
Source: Entropy (Basel). 2026 Sep 2;28(9):976. doi: 10.3390/e28090976 (PMC13605679; doi:10.3390/e28090976)
Supplement: Supplementary file 1 [file entropy-28-00976-s001.zip › entropy-4440869-supplementary.pdf]

entropy

MDPI

Article

Supplementary Materials: Contrasting Resilience Diagnostics in Route-Preserving Multimodal Transit Hypergraphs

Tian Gao and Ivan Blekanov *

Faculty of Applied Mathematics and Control Processes, St. Petersburg State University, St. Petersburg 199034, Russia; st082013@student.spbu.ru
* Correspondence: i.blekanov@spbu.ru

S1. Clustering validation

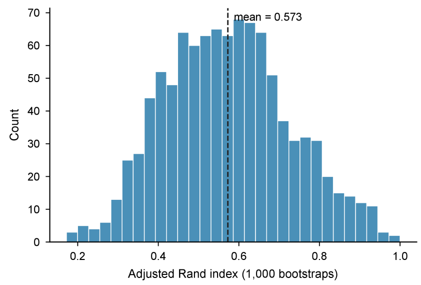

(**a**)

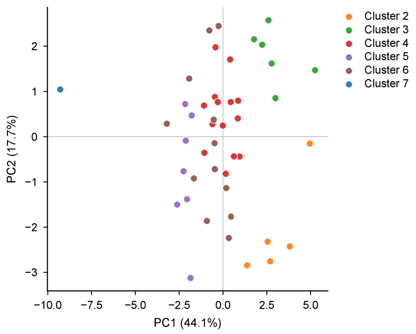

(**b**)

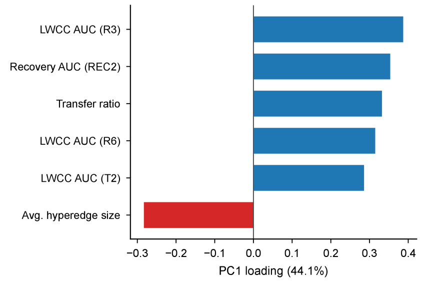

(**c**)

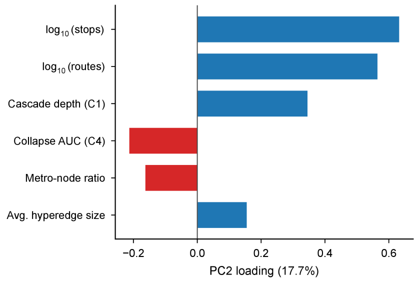

(**d**)

Figure S1. Clustering validation. (a) Bootstrap ARI distribution (1 000 resamples; dashed: mean ARI = 0.573). (b) PCA projection onto PC1–PC2 (61.8% variance), coloured by cluster. (c) Top-6 loadings on PC1 (44.1%). (d) Top-6 loadings on PC2 (17.7%).

Check for updates

Received: 1 July 2026
Revised: 20 August 2026
Accepted: 31 August 2026
Published: 2 September 2026

Copyright: © 2026 by the authors. Licensee MDPI, Basel, Switzerland. This article is an open access article distributed under the terms and conditions of the Creative Commons Attribution (CC BY) license.

S2. Outcome-informed exploratory-city structural signatures

Table S1. Structural signatures and recovery performance of the six outcome-informed exploratory-city groups used for computational coverage.

Cluster	Exploratory example	n	Structural type	z_{stop}	z_{route}	$z_{\bar{s}}$	$z_{\bar{d}_H}$	z_{metro}	z_{trans}	Rec. AUC
1	Guiyang	5	Compact high-PPCR	−1.14	−0.42	−1.12	+0.28	+1.35	+1.32	0.616
2	Chengdu	6	Large hub-dominant	+1.31	+1.57	−0.81	+0.55	+0.47	+0.39	0.605
3	Chuzhou	1	Minimal-metro outlier	−0.58	−0.41	+2.59	−0.73	−1.47	−1.45	0.198
4	Qingdao	14	Mid-size baseline	+0.43	+0.23	−0.15	−0.27	+0.29	+0.12	0.561
5	Shijiazhuang	7	Long-route sparse	−0.32	−1.00	+0.62	−0.80	−0.21	−0.88	0.535
6	Fuzhou	12	Low-transfer vulnerable	−0.09	−0.18	+0.07	+0.18	−0.44	−0.43	0.527

S3. Full six-predictor regression

The two size variables are total multimodal node count and total route-hyperedge count. VIFs are calculated from the standardized six-predictor matrix with an intercept ($n = 45$). They are not derived from one pairwise correlation in Figure 2a.

Table S2. Variance inflation factors (VIF) for the six structural predictors. Predictors with VIF > 10 exhibit multicollinearity, so the main text uses a reduced three-predictor specification.

Predictor	VIF
$\log_{10}$(stops)	470.7
$\log_{10}$(routes)	561.7
PPCR	2.35
Metro-node ratio	1.81
Avg. hyperdegree	52.7
Avg. hyperedge size	35.1

Table S3. Standardized regression coefficients (β) for six structural predictors across four resilience outcomes ($n = 45$). Bold: $p < 0.05$ for predictors with VIF < 10.

Predictor	Static retention (random node)	Cascade depth (C1)	Cascade breadth (C4)	Recovery (REC2)
$\log_{10}$(stops)	0.718	0.174	−0.051	1.471
$\log_{10}$(routes)	−0.722	1.030	0.046	−1.459
PPCR	**1.126**	**6.904**	**0.061**	0.651
Metro-node ratio	0.080	−6.111	−0.087	0.444
Avg. hyperdegree	0.071	−0.027	−0.002	0.144
Avg. hyperedge size	−0.016	−0.037	< 0.001	−0.031
R^2_{adj}	0.687	0.780	0.838	0.318
Q^2_{LOO}	0.536	0.716	0.642	0.048

S4. Structural-only clustering validation

Table S4. Comparison of the frozen structure-only (6 features) replication set and the outcome-informed (13 features) exploratory set ($K = 6$). ARI = 0.249; the sets share no cities; the 2×2 factorial decomposition gives consistent results in both sets.

Metric	Value	Interpretation
Adjusted Rand Index	0.249	Low agreement
Normalized Mutual Information	0.472	Moderate information overlap
City-set overlap	0/6	Zero shared cities
**13-feature outcome-informed exploratory set:**		
Guiyang, Chengdu, Chuzhou, Qingdao, Shijiazhuang, Fuzhou		
**6-feature frozen structure-only replication set:**		
Guangzhou, Shanghai, Tianjin, Xuzhou, Zhengzhou, Urumqi		
**Factorial decomposition replication (collapse fraction):**		
Rule effect (13-feature set)	0.424	
Rule effect (6-feature set)	0.429	Within 1.2%
Representation effect (both sets)	$\sim$0.05	Order of magnitude smaller

S5. Regression sensitivity: excluding Chuzhou

Table S5. Regression sensitivity to Chuzhou exclusion ($n = 44$ vs. $n = 45$). PPCR associations are largely stable; the cross-mode LCC vs. recovery Pearson correlation shows the largest leverage ($r = 0.834 \rightarrow 0.568$).

Analysis	Metric	Full sample	Excluding Chuzhou	Change
**Bivariate correlations:**				
PPCR vs. recovery AUC	Pearson r	0.513	0.479	$\lvert\Delta r\rvert = 0.034$
Cross-mode LCC vs. recovery AUC	Pearson r	0.834	0.568	$\lvert\Delta r\rvert = 0.266$
Cross-mode LCC vs. recovery AUC	Spearman ρ	0.570	0.540	$\lvert\Delta \rho\rvert = 0.030$
**Three-predictor regression coefficients (max change):**				
Static connectivity retention	β_{transfer}	1.126	1.089	−3.3%
Cascade depth	β_{transfer}	6.904	7.352	+6.5%
Cascade breadth	β_{transfer}	0.061	0.066	+7.8%
Recovery AUC	β_{transfer}	0.651	0.623	−4.3%
**Significance preservation:**				
Previously significant ($p < 0.05$)		17/18	17/18	No change
Previously non-significant		1/18	1/18	No change

S6. Comprehensive statistical tests with FDR correction

Table S6. Summary of major statistical tests with Benjamini–Hochberg FDR correction. Statistic types: r = Pearson correlation, F = omnibus regression F-test, t = regression coefficient t-test, W = Wilcoxon signed-rank. Of 18 tests, 17 remain below the FDR threshold at $q = 0.05$.

Category	Test description	Stat. type	Value	p_{raw}	p_{BH}
Bivariate	PPCR vs. static connectivity retention	r	0.807	2.1×10^{-11}	7.5×10^{-11}
Bivariate	$\log_{10}$(routes) vs. cascade depth	r	0.767	8.2×10^{-10}	2.5×10^{-9}
Bivariate	Route size vs. cascade breadth	r	−0.747	3.8×10^{-9}	8.5×10^{-9}
Bivariate	PPCR vs. T2 connectivity retention	r	0.531	1.7×10^{-4}	2.8×10^{-4}
Bivariate	PPCR vs. recovery AUC	r	0.513	3.2×10^{-4}	4.8×10^{-4}
Bivariate	Metro-node ratio vs. static connectivity retention	r	0.572	4.0×10^{-5}	7.2×10^{-5}
Regression (F)	6-predictor: static connectivity retention	F	17.09	1.9×10^{-9}	4.8×10^{-9}
Regression (F)	6-predictor: T2 connectivity retention	F	5.10	6.3×10^{-4}	8.7×10^{-4}
Regression (F)	6-predictor: cascade depth	F	26.99	2.8×10^{-12}	1.4×10^{-11}
Regression (F)	6-predictor: cascade breadth	F	39.07	8.8×10^{-15}	1.6×10^{-13}
Regression (F)	6-predictor: recovery AUC	F	4.41	1.8×10^{-3}	2.1×10^{-3}
Coefficient (t)	PPCR → static connectivity retention	t	4.82	2.3×10^{-5}	4.6×10^{-5}
Coefficient (t)	PPCR → T2 connectivity retention	t	2.07	0.046	0.048
Coefficient (t)	PPCR → cascade depth	t	3.48	1.3×10^{-3}	1.6×10^{-3}
Coefficient (t)	PPCR → cascade breadth	t	3.35	1.8×10^{-3}	2.1×10^{-3}
Coefficient (t)	PPCR → recovery AUC	t	1.31	0.197	0.197
Paired	Cascade depth: targeted > random (Wilcoxon)	W	1021	3.1×10^{-12}	1.4×10^{-11}
Paired	Recovery: hyperdegree > random (Wilcoxon)	W	1035	2.8×10^{-14}	2.6×10^{-13}

S7. Alternative-threshold cascade model comparison

Table S7. Cascade comparison at $\tau_{\text{RS}} = 0.2$, $\tau_{\text{NF}} = 0.3$ (10% random node removal, 50 repetitions). Route-support collapse equals the initial damage fraction (0.100) at both $\tau = 0.2$ and $\tau = 0.3$, indicating zero secondary amplification under diffuse damage.

	Route-support ($\tau = 0.2$)		Neighbour-fraction ($\tau = 0.3$)	
City	Collapse	Depth	Collapse	Depth
Guiyang	0.100	1.2	0.991	29.3
Chengdu	0.101	2.7	0.987	37.9
Chuzhou	0.100	1.4	0.988	42.1
Qingdao	0.100	1.5	0.994	41.1
Shijiazhuang	0.100	1.1	0.985	38.0
Fuzhou	0.100	1.7	0.986	36.5
Mean	0.100	1.6	0.988	37.5

S8. Stop-density confound control

Table S8. Partial correlations of PPCR with resilience outcomes, controlling for stop density (mean inter-stop distance) and system size ($\log_{10}$ stops). Mean inter-stop distance correlates only weakly with PPCR ($r = 0.334$, $p = 0.025$). All five partial correlations remain significant ($p < 0.01$) after controlling for density.

Outcome	r (zero-order)	r (ctrl: density)	r (ctrl: density + size)
Static retention (random node)	0.807	0.781	0.776
Static retention (betweenness)	0.531	0.507	0.499
Cascade depth (C1)	0.589	0.571	0.668
Cascade breadth (C4)	0.680	0.640	0.678
Recovery AUC (REC2)	0.513	0.469	0.458

S9. P-space representation comparison

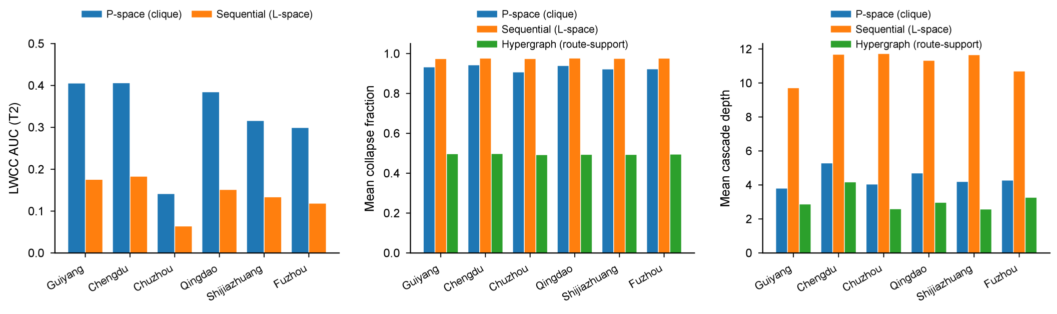

(**a**) (**b**) (**c**)

Figure S2. Exploratory P-space representation comparison (six outcome-informed cities). (a) LWCC AUC under targeted attack (T2). (b) Collapse fraction (C1, $\tau = 0.3$). (c) Cascade depth.

S10. Mechanical-exposure null model

Table S9. Correlation between PPCR and LWCC AUC under full random-removal pool vs. transfer-immune pool (all 45 cities). Immunizing transfer nodes strengthens rather than weakens the association.

Removal pool	Pearson r	p	Spearman ρ
Full pool (all nodes eligible)	0.807	2.1×10^{-11}	0.802
Transfer nodes immune	0.906	1.3×10^{-17}	0.948

S11. Cascade response to initial-damage intensity

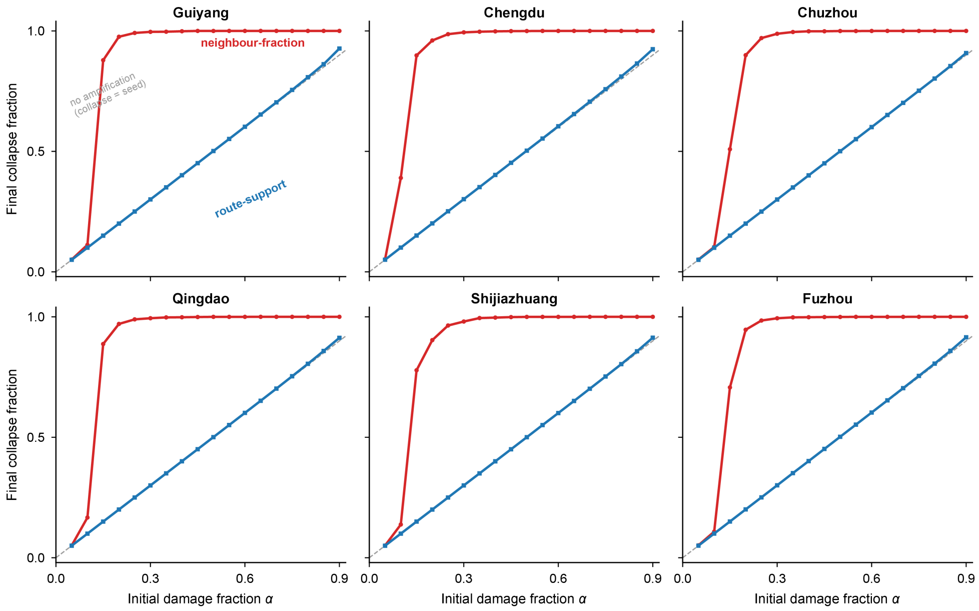

Figure S3. Exploratory collapse fraction vs. initial damage fraction f for six outcome-informed cities ($\tau = 0.3$, 20 repetitions per f). Blue squares: route-support rule; red circles: neighbour-fraction rule (P-space clique); dashed: no-amplification reference (collapse $= f$).

S12. Intercorrelation structure of the resilience outcomes

We tested whether static retention, cascade propagation, and recovery represent distinct resilience dimensions. We computed Pearson correlations among the five resilience outcomes across all 45 cities (Table S10) and applied PCA to the 45×5 outcome matrix (Fig. S4). Off-diagonal correlations are moderate (mean $|r| = 0.517$, median $= 0.508$), ranging from a high of $r = 0.857$ (static random vs. recovery AUC) to a non-significant low of $r = 0.217$ (static targeted vs. cascade breadth, $p = 0.15$). The first principal component explains only 62.6% of the total variance; three components are required to reach the 90% threshold. If the outcomes collapsed onto a single "resilience" axis, PC1 would approach 100%. The retention of substantial variance on PC2 (19.2%) and PC3 (10.9%) indicates that the outcomes occupy a multi-dimensional space. The component loadings (Table S12) show that the two cascade outcomes load with the opposite sign to the static and recovery outcomes on PC2 and dominate PC3, so cascade propagation occupies a direction largely orthogonal to static retention and recovery.

Table S10. Pearson correlation matrix among the five resilience outcomes ($n = 45$ cities). Significance: $^{*}p < 0.05$, $^{**}p < 0.01$, $^{***}p < 0.001$. Off-diagonal $|r|$: mean $= 0.517$, median $= 0.508$, max $= 0.857$, min $= 0.217$.

	Static (rand.)	Static (targ.)	Casc. depth	Casc. breadth	Recovery
Static (random)	1.000	0.712***	0.543***	0.634***	0.857***
Static (targeted)	0.712***	1.000	0.281	0.217	0.688***
Cascade depth	0.543***	0.281	1.000	0.473**	0.367*
Cascade breadth	0.634***	0.217	0.473**	1.000	0.394**
Recovery AUC	0.857***	0.688***	0.367*	0.394**	1.000

Table S11. PCA of the 45 × 5 z-scored resilience-outcome matrix. Three components are needed to reach ≥ 90% cumulative variance; PC1 explains only 62.6%, rejecting a single-axis collapse.

	PC1	PC2	PC3	PC4	PC5
Explained variance	0.626	0.192	0.109	0.058	0.015
Cumulative	0.626	0.818	0.927	0.985	1.000
Eigenvalue	3.129	0.962	0.545	0.289	0.075

Table S12. Loadings of the five resilience outcomes on the first five principal components of the 45 × 5 z-scored outcome matrix.

Outcome	PC1	PC2	PC3	PC4	PC5
Static (random)	−0.547	+0.050	−0.121	−0.119	+0.818
Static (targeted)	−0.428	+0.533	+0.163	+0.684	−0.195
Cascade depth	−0.365	−0.529	+0.759	−0.026	−0.103
Cascade breadth	−0.378	−0.577	−0.611	+0.282	−0.267
Recovery AUC	−0.491	+0.317	−0.100	−0.662	−0.459

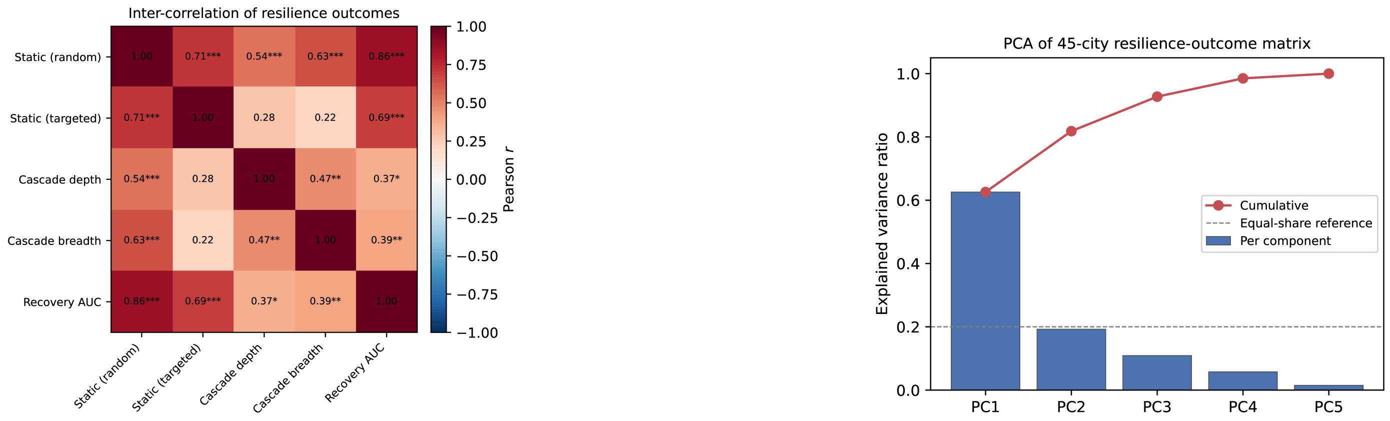

(**a**) (**b**)

Figure S4. Intercorrelation structure of the resilience outcomes ($n = 45$). (a) Pearson correlation heatmap (stars: significance levels). (b) Scree plot of the PCA explained variance; dashed: equal-share reference.

S13. Cross-threshold stability of the cascade-depth correlations

The two structural correlates that drive the cascade-depth findings in the main text—route count ($r = 0.767$ with cascade depth) and PPCR ($r = 0.589$)—are reported at the 200 m walk-transfer threshold. To confirm that these associations are not artifacts of that single threshold, we decompose their stability across the four network versions ($\delta \in \{0, 100, 200, 300\}$ m) using data already on disk, without re-running the 45-city cascade simulations. The two correlates behave differently and are treated separately.

The stronger correlate, route count, is threshold-invariant by construction: walk-transfer edges add transfer relationships between existing route-stops but create no new routes, so $\log_{10}(\text{routes})$ is identical for all 45 cities across all four versions (Spearman $\rho = 1.000$ in every pairwise comparison; Table S13, Panel A). Recomputing cascade depth under each threshold for the six outcome-informed exploratory cities shifts it by at most 1.1% per city (Panel B), and the route-count – cascade-depth correlation on that set holds at $r = 0.917$–0.922 across all four thresholds. The dominant cascade-depth correlate therefore cannot be a consequence of how walk-transfers are defined.

PPCR changes in level as δ changes. The city mean rises from 0.014 at $\delta = 0$ to 0.079 at $\delta = 300$ m. City rankings remain stable at 100 m ($\rho = 0.974$) and 300 m ($\rho = 0.996$), relative to 200 m. The name-match-only definition is different and gives $\rho = 0.428$. This pattern is consistent with the ranking sensitivity of cascade depth (Table 5). The transfer

definition changes individual city orderings more than the structural correlations. We did not recompute the full-sample PPCR–depth correlation at every alternate threshold because that would require rerunning all cascades.

These checks test geometric-link sensitivity. They do not validate links against observed transfers, station maps, GTFS/NeTEx fields, smart-card records, or walking trajectories.

Table S13. Cross-threshold stability of cascade-depth correlations. Panel A: Spearman ρ of structural predictors vs. the 200 m reference ($n = 45$). Panel B: exploratory cascade depth (C1, $\tau = 0.2$) per walk-transfer threshold for six outcome-informed cities; maximum drift 1.10%.

**Panel A. Predictor rank stability (Spearman ρ vs. 200 m, $n = 45$):**				
Predictor	$\delta = 0$	100 m	200 m	300 m
$\log_{10}$(routes)	1.000	1.000	—	1.000
PPCR	0.428	0.974	—	0.996
**Panel B. Cascade depth recomputed per threshold (6 outcome-informed exploratory cities):**				
City	$\delta = 0$	100 m	200 m	300 m
Guiyang	2.86	2.89	2.88	2.87
Chengdu	4.17	4.17	4.18	4.17
Chuzhou	2.60	2.59	2.60	2.62
Qingdao	2.98	2.98	2.98	2.98
Shijiazhuang	2.55	2.57	2.57	2.56
Fuzhou	3.25	3.27	3.27	3.28
$r\,[\log_{10}$(routes), depth]	0.922	0.920	0.919	0.917

S14. Outcome-informed exploratory-city disruption trajectories

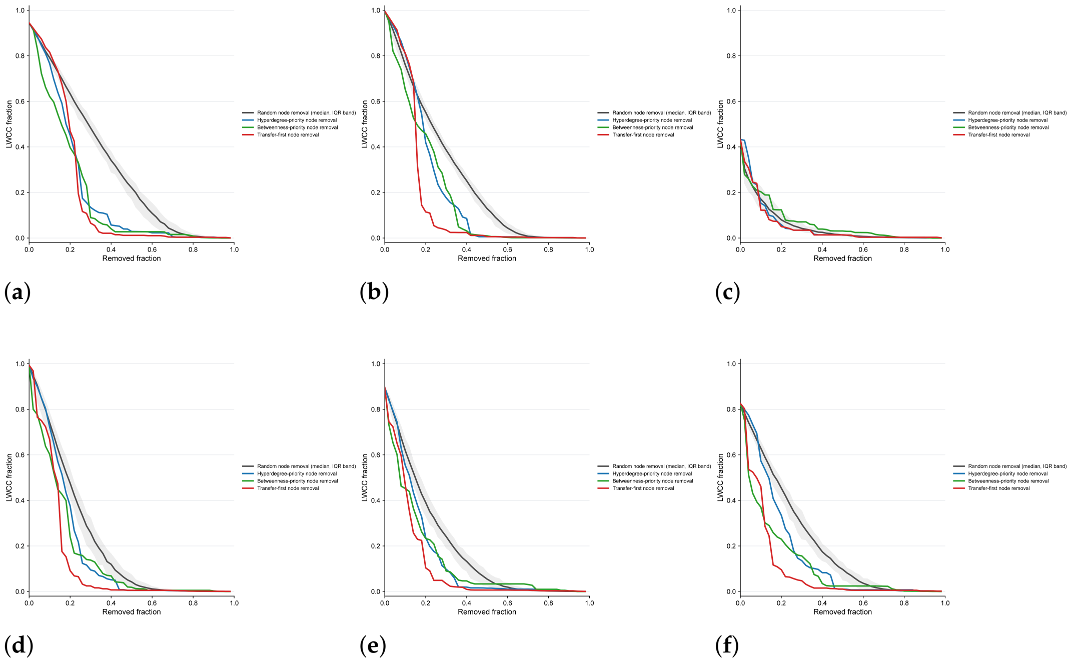

Figure S5. Exploratory static attack trajectories for the six outcome-informed cities. LWCC fraction vs. attacked node fraction under random (grey band: median and IQR), hyperdegree-priority (blue), betweenness-priority (green), and transfer-first (red) removal.

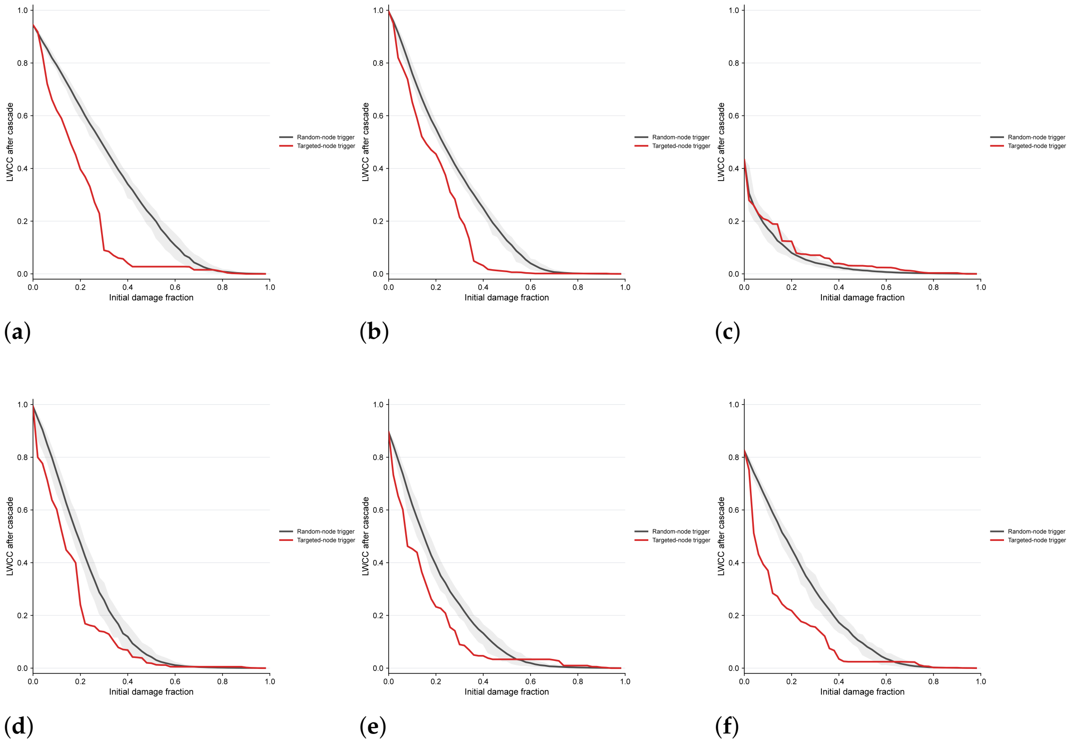

Figure S6. Exploratory cascade trajectories for the six outcome-informed cities. LWCC fraction vs. initial damage fraction under random-node (grey) and targeted-node (red) triggering at $\tau = 0.2$.

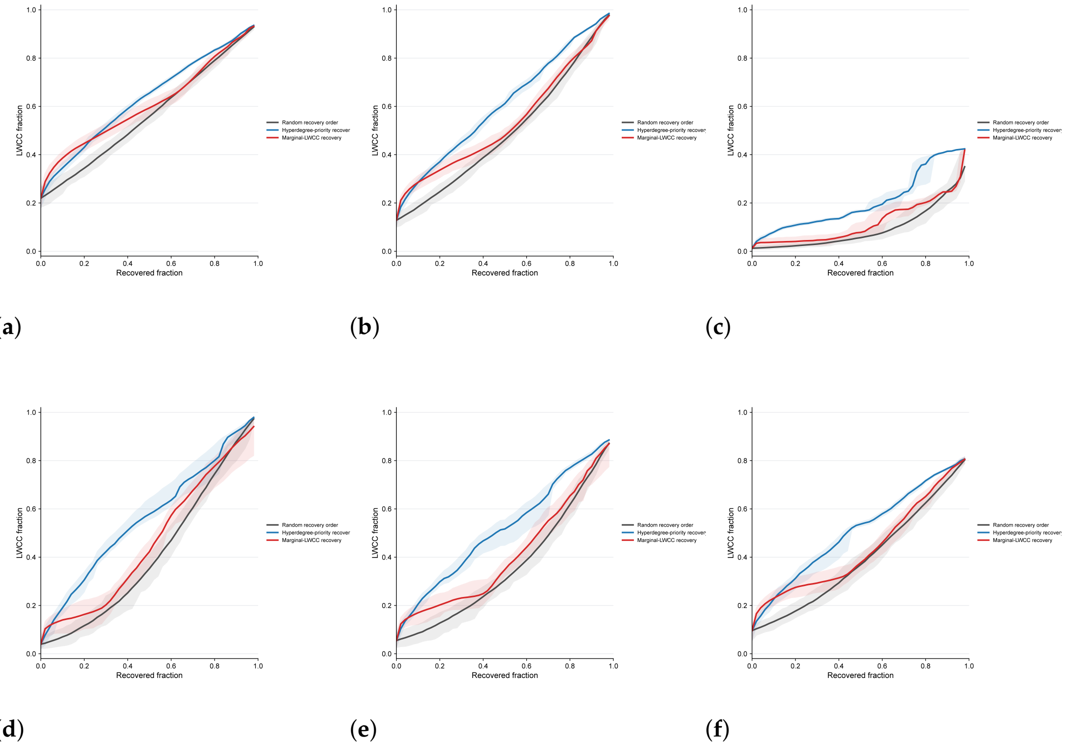

Figure S7. Exploratory recovery trajectories for the six outcome-informed cities. LWCC fraction vs. restored node fraction under random (grey), hyperdegree-priority (blue), and marginal-LWCC (green) recovery, starting from identical random-node damage.

S15. T2 vs T3 damage gap by metro-node ratio

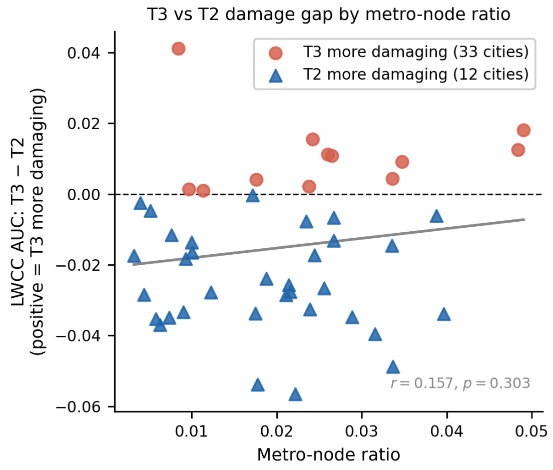

Figure S8. (T3 − T2) LWCC AUC gap vs. metro-node ratio across 45 cities. Positive: T3 more damaging; negative: T2 more damaging. Line: OLS fit.

S16. Spatial autocorrelation of OLS residuals (Moran's I)

We tested the OLS residuals for spatial autocorrelation using Moran's I. The weights are inverse great-circle distances, row-standardized across 45 cities. Each test uses 999 permutations. No result is significant ($p \geq 0.147$; Table S14), so geographic clustering does not explain the reported associations.

Table S14. Moran's I spatial autocorrelation test on OLS residuals for four resilience outcomes ($n = 45$ cities, 999 permutations, inverse great-circle-distance weight matrix row-standardised).

Outcome	Regression	Moran's I	p (permutation)
LWCC AUC (random node, R1)	PPCR	0.020	0.673
Cascade depth (C1)	PPCR	−0.009	0.864
Collapse breadth (C4)	PPCR	0.061	0.147
Recovery AUC (REC2)	PPCR	−0.014	0.771

S17. Route-support cascade midpoint f_c across exploratory cities

The logistic midpoint $f_c(\tau)$ is the initial-damage fraction at which collapse reaches half of its asymptotic value. We fit $\Gamma(f) = [1 + \exp(-k(f - f_c))]^{-1}$ separately for each city at $\tau \in \{0.2, 0.4, 0.6\}$. Table S15 reports the fitted values. Across all 18 city–τ combinations, the mean is 0.498 ± 0.002 and the range is 0.494–0.500.

Table S15. Logistic midpoint f_c of the route-support cascade for the six outcome-informed exploratory cities at three tolerance values. Fit: $\Gamma(f) = [1 + \exp(-k(f - f_c))]^{-1}$; C1/C2/C3 attack curves, 100 Monte Carlo repetitions each. Overall: mean = 0.498, SD = 0.002, range $[0.494, 0.500]$.

City	$\tau = 0.2$	$\tau = 0.4$	$\tau = 0.6$	Mean	SD
Chengdu	0.494	0.496	0.498	0.496	0.002
Chuzhou	0.499	0.499	0.500	0.499	0.001
Fuzhou	0.496	0.498	0.499	0.498	0.001
Guiyang	0.495	0.497	0.499	0.497	0.002
Qingdao	0.498	0.499	0.500	0.499	0.001
Shijiazhuang	0.498	0.499	0.500	0.499	0.001
Overall mean	0.497	0.498	0.499	0.498	0.002

S18. Sensitivity to route-viability and tolerance assumptions

The route-support rule is a structural counterfactual, not a calibrated operational model. We test sensitivity in six outcome-informed exploratory cities on the same attack

grid ($f = 0$–0.9, step 0.1), with matched seeds and 20 repetitions. Panel A of Table S16 varies route viability at $\tau = 0.2$. Panel B varies τ under the two-stop condition. Stricter proportional viability conditions increase depth and collapse breadth. Changing τ has a smaller effect on collapse breadth.

Table S16. Exploratory sensitivity of route-support cascade outcomes across six outcome-informed cities. Values are means across cities over the full attack curve. Fixed-2: a route remains viable when at least two stops survive.

Condition	Mean cascade depth	Mean collapse fraction	LWCC AUC
**Panel A. Route-viability condition ($\tau = 0.2$)**			
Fixed-2	1.681	0.451	0.2069
$\alpha = 0.1$	1.758	0.453	0.2069
$\alpha = 0.2$	2.185	0.464	0.2068
$\alpha = 0.3$	2.578	0.487	0.2064
$\alpha = 0.5$	3.204	0.560	0.1995
$\alpha = 0.7$	3.625	0.683	0.1626
**Panel B. Tolerance threshold (fixed-2)**			
$\tau = 0.1$	1.671	0.4512	0.2069
$\tau = 0.2$	1.681	0.4512	0.2069
$\tau = 0.3$	1.725	0.4512	0.2069
$\tau = 0.4$	1.782	0.4513	0.2069
$\tau = 0.5$	1.804	0.4513	0.2069
$\tau = 0.6$	2.287	0.4523	0.2068
$\tau = 0.7$	2.545	0.4531	0.2066
$\tau = 0.8$	3.005	0.4545	0.2065

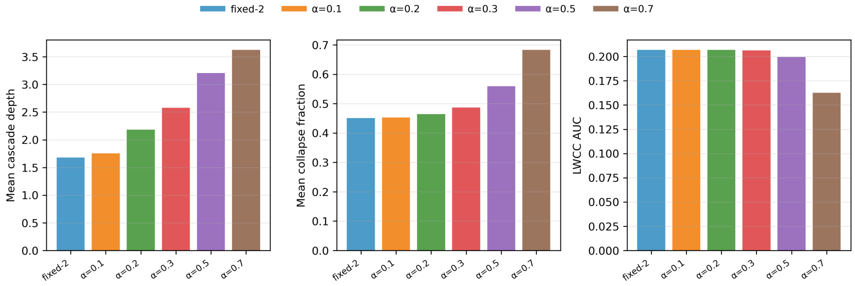

Figure S9. Exploratory sensitivity to the route-viability condition across six outcome-informed cities. Each panel summarizes full-curve outcomes at $\tau = 0.2$.

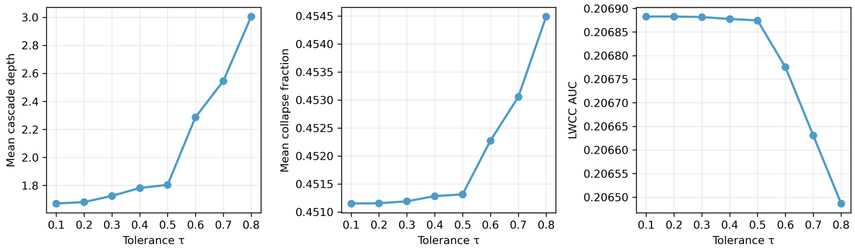

Figure S10. Exploratory sensitivity to the tolerance threshold τ under the fixed two-stop route-viability condition. Each line denotes one outcome-informed city; values summarize full-curve outcomes.

S19. Repetition-based uncertainty intervals

Table S17 reports empirical 95% intervals across 20 matched repetitions. It includes the fixed condition and two proportional alternatives. These intervals quantify Monte Carlo variability under a fixed structural rule; they do not calibrate the model to observed disruptions.

Table S17. Repetition-based uncertainty intervals for route-support cascade sensitivity at $\tau = 0.2$. Values are mean [2.5th percentile, 97.5th percentile] across 20 matched repetitions and summarize the full attack curve.

City	Condition	Cascade depth	Collapse fraction
Guiyang	Fixed-2	1.550 [1.400, 1.700]	0.4514 [0.4510, 0.4518]
Guiyang	$\alpha = 0.3$	2.390 [2.100, 2.800]	0.4859 [0.4837, 0.4878]
Guiyang	$\alpha = 0.7$	3.490 [2.748, 4.452]	0.6860 [0.6717, 0.7135]
Chengdu	Fixed-2	1.940 [1.900, 2.100]	0.4519 [0.4517, 0.4521]
Chengdu	$\alpha = 0.3$	3.085 [2.647, 3.610]	0.4846 [0.4836, 0.4855]
Chengdu	$\alpha = 0.7$	4.310 [3.795, 5.162]	0.6759 [0.6709, 0.6823]
Chuzhou	Fixed-2	1.480 [1.300, 1.700]	0.4505 [0.4504, 0.4507]
Chuzhou	$\alpha = 0.3$	2.250 [1.847, 2.600]	0.4889 [0.4850, 0.4935]
Chuzhou	$\alpha = 0.7$	2.980 [2.590, 3.458]	0.6855 [0.6773, 0.6949]
Qingdao	Fixed-2	1.745 [1.600, 1.900]	0.4510 [0.4509, 0.4511]
Qingdao	$\alpha = 0.3$	2.700 [2.348, 3.157]	0.4863 [0.4840, 0.4883]
Qingdao	$\alpha = 0.7$	3.915 [3.147, 4.605]	0.6827 [0.6764, 0.6904]
Shijiazhuang	Fixed-2	1.545 [1.300, 1.853]	0.4508 [0.4506, 0.4511]
Shijiazhuang	$\alpha = 0.3$	2.365 [2.047, 2.752]	0.4867 [0.4850, 0.4883]
Shijiazhuang	$\alpha = 0.7$	3.370 [2.695, 4.572]	0.6842 [0.6765, 0.6943]
Fuzhou	Fixed-2	1.825 [1.647, 1.952]	0.4514 [0.4511, 0.4516]
Fuzhou	$\alpha = 0.3$	2.675 [2.395, 3.357]	0.4870 [0.4857, 0.4885]
Fuzhou	$\alpha = 0.7$	3.685 [2.842, 4.753]	0.6843 [0.6740, 0.6966]

S20. Matched route-support reconstruction across representations

We tested whether representation changes the result once route membership is preserved. The native hypergraph was compared with L-space and P-space supplied with the same route-incidence context. The experiment used 10% random damage, $\tau = 0.30$, and 50 matched repetitions in six cities. All three implementations produced identical collapse fractions and depths.

Table S18. Per-city matched route-support reconstruction results. Values are means over 50 repetitions; the three route-support representations were numerically identical.

City	Mean collapse fraction	Mean cascade depth
Guiyang	0.1001	1.24
Chengdu	0.1005	2.58
Chuzhou	0.1000	1.14
Qingdao	0.1001	1.46
Shijiazhuang	0.1000	1.06
Fuzhou	0.1001	1.60

S21. Cross-mode endpoints under transfer-edge removal

We computed LWCC, cross-mode LCC, and cross-mode reachability under random transfer-edge removal in six cities. Each curve uses 20 repetitions and 11 removal fractions. Cross-mode reachability is the fraction of sampled ordered cross-mode pairs connected by a directed path. The cross-mode endpoints decline more than LWCC, so overall connectivity can conceal loss of multimodal integration.

Table S19. AUC of complementary structural endpoints under R3 transfer-edge removal.

City	LWCC AUC	Cross-mode LCC AUC	Cross-mode reachability AUC
Chengdu	0.979	0.933	0.916
Chuzhou	0.433	0.411	0.401
Shijiazhuang	0.857	0.818	0.810
Fuzhou	0.814	0.775	0.762
Guiyang	0.943	0.897	0.876
Qingdao	0.944	0.904	0.879
Median	0.900	0.858	0.843

S22. Bootstrap stability of primary contrasts selected during the experimental stage

During the experimental analysis, we selected three primary contrasts before examining the bootstrap draws. H1 concerns PPCR and random-damage LWCC AUC. H2 concerns PPCR and route-support depth. H3 compares targeted and random depth at $\tau = 0.2$. We resampled the 45 cities 5,000 times and calculated percentile 95% intervals. The intervals describe sample stability, not causal effects or uncertainty in a new city population.

Table S20. Bootstrap stability intervals for the three primary contrasts selected during the experimental stage ($n = 45$ cities; 5,000 city-level resamples).

Hypothesis	Contrast	Estimate	95% interval	Statistic
H1	PPCR–LWCC AUC (random node)	0.807	[0.688, 0.895]	Pearson r
H2	PPCR–cascade depth	0.589	[0.289, 0.778]	Pearson r
H3	Targeted − random cascade depth	0.475	[0.380, 0.577]	Mean difference

S23. Model-selection uncertainty

We repeated five-fold cross-validated LASSO on 2,000 city-level bootstrap samples. Predictors were standardized within each resample. A coefficient was selected when its absolute value exceeded 10^{-8}. The reduced model contains log stop count, PPCR, and metro-node ratio. The full model adds log route count, average hyperdegree, and average route size.

Table S21. LASSO selection frequencies and standardized coefficient intervals across 2,000 bootstrap samples.

Outcome	Stops	Routes	PPCR	Metro ratio	Hyperdegree	Route size
LWCC AUC (R3)	0.518	0.523	0.999	0.695	0.794	0.863
Cascade depth (C1)	0.639	0.885	0.915	0.794	0.460	0.990
Collapse breadth (C4)	0.994	0.360	0.997	0.949	0.958	0.969

Table S22. Bootstrap standardized LASSO coefficient intervals for PPCR (2,000 resamples).

Outcome	Selection frequency	Median coefficient	95% interval
LWCC AUC (R3)	0.999	0.639	[0.356, 1.148]
Cascade depth (C1)	0.915	0.329	[0.000, 0.642]
Collapse breadth (C4)	0.997	0.299	[0.152, 0.961]

Table S23. Bootstrap comparison of six-predictor and reduced three-predictor OLS specifications. Values are full-model R^2 minus reduced-model R^2 (5,000 resamples).

Outcome	Mean difference	2.5th percentile	97.5th percentile
LWCC AUC (R3)	0.085	0.006	0.215
Cascade depth (C1)	0.125	0.034	0.270
Collapse breadth (C4)	0.325	0.167	0.542

No independent city set or later release of CPTOND-2025 was available for external validation. Accordingly, the revised manuscript reports bootstrap stability and leave-one-city-out performance as internal checks, and does not claim transportability or causal generalisation beyond the observed 45-city cross-section.

S25. Pre-disruption freeze audit for analysis-set selection

We audited city-set selection to remove outcome information from the confirmatory replication. Structure-only clustering uses six pre-simulation descriptors: log stop count, log route count, PPCR, metro-node ratio, average hyperdegree, and average route size. With $K = 6$, centroid-nearest selection gives Guangzhou, Shanghai, Tianjin, Xuzhou, Zhengzhou, and Urumqi. The 13-feature outcome-informed partition is reported separately as the exploratory set.

Table S24. Frozen structure-only replication set and separation from the outcome-informed exploratory set.

Selection role	Features used before simulation	Selected cities
Confirmatory (frozen)	Six structural descriptors only	Guangzhou; Shanghai; Tianjin; Xuzhou; Zhengzhou; Urumqi
Descriptive (outcome-informed)	Six structural + seven resilience summaries	Guiyang; Chengdu; Chuzhou; Qingdao; Shijiazhuang; Fuzhou

The two sets share no city. In the frozen set, the rule effect on collapse is 0.429, compared with 0.424 in the outcome-informed set. The difference is 1.2%, and the representation effect remains about 0.05. Thus, outcome-informed selection does not generate the qualitative model comparison.

S26. Transfer-rule network audit

We audited the inferred cross-modal edge set under the name-only, 100 m, 200 m and 300 m definitions. These are structural sensitivity checks, not behavioural validation of usable passenger transfers.

Table S25. Geographically admissible cross-city transfer-rule audit ($n = 45$ cities). For every rule, including name matches, only pairs within that rule's distance threshold are retained; this is the same edge definition shown in Fig. S11. Jaccard similarity and overlap are calculated relative to the 200 m reference edge set.

Rule	Mean edges	Median edges	Mean Jaccard	Mean overlap	PPCR rank ρ
Name-only (200 m cap)	15.1	9	0.074	0.074	0.428
100 m	100.1	64	0.371	0.371	0.974
200 m (reference)	269.1	167	1.000	1.000	1.000
300 m	438.4	268	0.631	1.000	0.996

S27. Dense sampling audit for Fig. 8b

We reran transfer-pair removal from 0.90 to 1.00 in steps of 0.01. The projected-network definition is unchanged, and the run uses a fixed seed. The endpoint transition is discrete: several cities retain the same cross-mode LCC until their final transfer pairs disappear. Table values are means across five repetitions. A zero standard deviation occurs when rounding selects the same number of removed pairs in every repetition.

Table S26. Dense-grid cross-mode LCC values in the 0.90–1.00 transfer-pair-removal interval (selected grid points; full dense-grid data can be regenerated using the public reproducibility repository).

City	0.90	0.95	0.99	1.00
Chuzhou	0.433	0.433	0.000	0.000
Shaoxing	0.983	0.983	0.983	0.000
Dongguan	0.998	0.998	0.000	0.000
Fuzhou	0.811	0.808	0.807	0.000
Guiyang	0.943	0.943	0.943	0.000
Shenzhen	0.974	0.974	0.972	0.000

S28. Baseline connectivity audit

Table S27. Baseline weak-connectivity and cross-mode audit across transfer rules. Values are city means; minimum values are the lowest city-level observation.

Rule	Mean components	Mean LWCC	Min LWCC	Mean cross-mode reach.	Min cross-mode reach.
Name-only	9.04	0.950	0.433	0.948	0.431
100 m	7.84	0.954	0.433	0.953	0.431
200 m	6.98	0.954	0.433	0.953	0.431
300 m	6.84	0.954	0.433	0.953	0.431

All 45 cities contain at least one mixed bus–metro component under the 200 m rule. Chuzhou is the lowest-coupling case: 15 inferred transfer edges, seven weak components, LWCC 0.433, and cross-mode reachability 0.431. We interpret the disconnected remainder as a coverage and coupling diagnostic, not an operational interruption. The simulations do not artificially connect the networks.

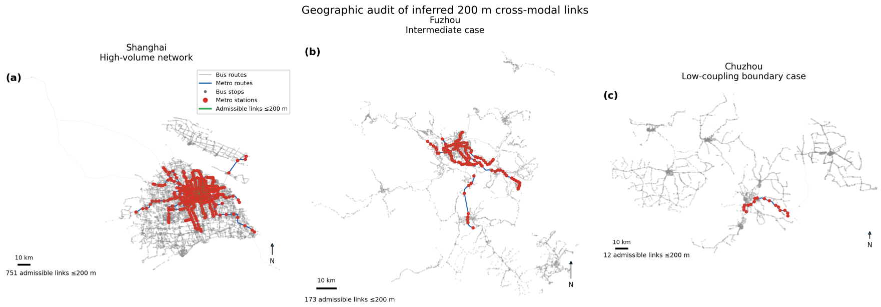

Figure S11. Geographic audit of inferred 200 m cross-modal links for three structural cases. Grey lines and points show the CPTOND bus layer; blue lines and red points show the metro layer; green segments are inferred bus–metro proximity links. The panels illustrate network coverage and inferred coupling structure, not validated pedestrian paths or observed passenger transfers.

S29. Disruption-event semantics

The two-stop condition is the minimal-connectivity convention used in this study. If exactly two ordered stops remain, the route is retained as a minimal surviving segment connecting two distinct stop nodes. If fewer than two stops remain, the route cannot provide a connecting relation in the projected network and is marked non-viable. This convention prevents a single surviving stop from being counted as a functioning route; it is not a claim that a real service can operate with exactly two stops.

At the node level, the viable-route fraction is evaluated synchronously. If three metro routes serve a station and two become non-viable, the fraction is $1/3$. The station is removed under the structural rule when $\tau > 1/3$ and remains supported when $\tau \leq 1/3$. This represents loss of route-based structural support, not necessarily physical station closure. The rule is deterministic and differs from an epidemic model, which typically uses transmission probabilities, infection states, and recovery transitions.

Table S28. Structural semantics of the disruption protocol.

Event	Model operation	What remains present	Interpretation
Node removal	Remove the node from every incident ordered route sequence	Other nodes and route records remain	Initial controlled damage
Route non-viability	Mark a route inactive when it fails the selected minimal-connectivity condition	Remaining stops are not removed directly	Route-support loss, not observed service closure
Node support failure	Remove a node when its viable-route fraction falls below τ in the next synchronous round	Other nodes update from the previous round	Structural support loss
Transfer-edge removal	Remove the bus–metro edge only	Both endpoint nodes remain	Loss of inferred cross-mode coupling
Cascade round	Apply all new failures synchronously	State from round t is used for round $t+1$	Controlled structural propagation
